# Sex differences in circulating microRNA profiles in heart failure with preserved and reduced ejection fraction

**DOI:** 10.1093/ehjopen/oeag073

**Published:** 2026-05-13

**Authors:** Genri Numata, Shun Nakamura, Hiroyuki Tokiwa, Akashi Taguchi, Masayuki Toyoda, Takashi Kohno, Hisataka Maki, Yasuyuki Shiraishi, Shinsuke Takeuchi, Ayumi Goda, Kyohei Daigo, Natsuko Yamamoto, Yusuke Adachi, Kosuke Yabe, Takao Kato, Yukio Hiroi, Norihiro Kato, Takuya Kawahara, Kazutaka Ueda, Eisuke Amiya, Masaru Hatano, Hideo Fujita, Masaki Ieda, Tetsuo Sasano, Norihiko Takeda, Takahide Kohro, Yasushi Hirota, Issei Komuro, Youichiro Wada, Eiki Takimoto

**Affiliations:** Isotope Science Center, The University of Tokyo, 2-11-16 Yayoi, Bunkyo-ku, Tokyo 113-0032, Japan; Department of Cardiovascular Medicine, The University of Tokyo Hospital, 7-3-1 Hongo, Bunkyo-ku, Tokyo 113-8655, Japan; Department of Cardiovascular Medicine, The University of Tokyo Hospital, 7-3-1 Hongo, Bunkyo-ku, Tokyo 113-8655, Japan; Department of Cardiovascular Medicine, Institute of Science Tokyo, 1-5-45 Yushima, Bunkyo-ku, Tokyo 113-8510, Japan; Department of Cardiovascular Medicine, The University of Tokyo Hospital, 7-3-1 Hongo, Bunkyo-ku, Tokyo 113-8655, Japan; Isotope Science Center, The University of Tokyo, 2-11-16 Yayoi, Bunkyo-ku, Tokyo 113-0032, Japan; Department of Cardiovascular Medicine, The University of Tokyo Hospital, 7-3-1 Hongo, Bunkyo-ku, Tokyo 113-8655, Japan; Department of Cardiovascular Medicine, Kyorin University School of Medicine, 6-20-2 Shinkawa, Mitaka-shi, Tokyo 181-8611, Japan; Division of Cardiovascular Medicine, Saitama Medical Center, Jichi Medical University, 1-847 Amanumacho, Omiya-ku, Saitama-shi, Saitama 330-8503, Japan; Department of Cardiology, Keio University School of Medicine, 35 Shinanomachi, Shinjuku-ku, Tokyo 160-8582, Japan; Department of Cardiovascular Medicine, Kyorin University School of Medicine, 6-20-2 Shinkawa, Mitaka-shi, Tokyo 181-8611, Japan; Department of Cardiovascular Medicine, Kyorin University School of Medicine, 6-20-2 Shinkawa, Mitaka-shi, Tokyo 181-8611, Japan; Department of Cardiology, Keio University School of Medicine, 35 Shinanomachi, Shinjuku-ku, Tokyo 160-8582, Japan; Department of Cardiovascular Medicine, The University of Tokyo Hospital, 7-3-1 Hongo, Bunkyo-ku, Tokyo 113-8655, Japan; Division of Cardiovascular Medicine, Saitama Medical Center, Jichi Medical University, 1-847 Amanumacho, Omiya-ku, Saitama-shi, Saitama 330-8503, Japan; Department of Cardiovascular Medicine, The University of Tokyo Hospital, 7-3-1 Hongo, Bunkyo-ku, Tokyo 113-8655, Japan; Horikiri Central Hospital, 7-4-4 Horikiri, Katsushika-ku, Tokyo 124-0006, Japan; Department of Cardiovascular Biology and Medicine, Juntendo University Graduate School of Medicine, 2-1-1 Hongo, Bunkyo-ku, Tokyo 113-8421, Japan; Department of Cardiology, National Center for Global Health and Medicine, Japan Institute for Health Security, 1-21-1 Toyama, Shinjuku-ku, Tokyo 162-8655, Japan; Medical Genomics Center, National Institute of Global Health and Medicine, Japan Institute for Health Security, 1-21-1 Toyama, Shinjuku-ku, Tokyo 162-8655, Japan; Clinical Research Promotion Center, The University of Tokyo Hospital, 7-3-1 Hongo, Bunkyo-ku, Tokyo 113-8655, Japan; Department of Cardiovascular Medicine, The University of Tokyo Hospital, 7-3-1 Hongo, Bunkyo-ku, Tokyo 113-8655, Japan; International University of Health and Welfare, 4-1-26 Akasaka, Minato-ku, Tokyo 107-8402, Japan; Department of Cardiovascular Medicine, The University of Tokyo Hospital, 7-3-1 Hongo, Bunkyo-ku, Tokyo 113-8655, Japan; Department of Cardiovascular Medicine, The University of Tokyo Hospital, 7-3-1 Hongo, Bunkyo-ku, Tokyo 113-8655, Japan; Advanced Medical Center for Heart Failure, The University of Tokyo Hospital, 7-3-1 Hongo, Bunkyo-ku, Tokyo 113-8655, Japan; Division of Cardiovascular Medicine, Saitama Medical Center, Jichi Medical University, 1-847 Amanumacho, Omiya-ku, Saitama-shi, Saitama 330-8503, Japan; Department of Cardiology, Keio University School of Medicine, 35 Shinanomachi, Shinjuku-ku, Tokyo 160-8582, Japan; Department of Cardiovascular Medicine, Institute of Science Tokyo, 1-5-45 Yushima, Bunkyo-ku, Tokyo 113-8510, Japan; Department of Cardiovascular Medicine, The University of Tokyo Hospital, 7-3-1 Hongo, Bunkyo-ku, Tokyo 113-8655, Japan; Department of Clinical Informatics, Jichi Medical University School of Medicine, 3311-1 Yakushiji, Shimotsuke-shi, Tochigi 329-0498, Japan; Department of Obstetrics and Gynecology, Graduate School of Medicine, The University of Tokyo, 7-3-1 Hongo, Bunkyo-ku, Tokyo 113-8655, Japan; International University of Health and Welfare, 4-1-26 Akasaka, Minato-ku, Tokyo 107-8402, Japan; Department of Frontier Cardiovascular Science, Graduate School of Medicine, The University of Tokyo, 7-3-1 Hongo, Bunkyo-ku, Tokyo 113-8655, Japan; Isotope Science Center, The University of Tokyo, 2-11-16 Yayoi, Bunkyo-ku, Tokyo 113-0032, Japan; Department of Cardiovascular Medicine, The University of Tokyo Hospital, 7-3-1 Hongo, Bunkyo-ku, Tokyo 113-8655, Japan; Medical Genomics Center, National Institute of Global Health and Medicine, Japan Institute for Health Security, 1-21-1 Toyama, Shinjuku-ku, Tokyo 162-8655, Japan; Division of Cardiology, Department of Medicine, The Johns Hopkins Medical Institutions, 720 Rutland Ave., Baltimore, MD 21205, USA

**Keywords:** Heart failure, HFpEF, HFrEF, Circulating microRNA, Sex differences, Sex-stratified analysis

## Abstract

**Aims:**

Circulating microRNAs (miRNAs) are informative markers of heart failure (HF); however, sex differences in HF-associated miRNA changes remain unclear. We performed sex-stratified analyses to assess whether HF-associated circulating miRNA changes differ by sex in HF with preserved ejection fraction (HFpEF) and HF with reduced ejection fraction (HFrEF).

**Methods and results:**

In this prospective multicentre cohort study in Japan, we analysed 235 participants, including 82 controls (41 males and 41 females), 76 patients with HFpEF (39 males and 37 females), and 77 patients with HFrEF (54 males and 23 females). Unbiased whole-blood miRNA sequencing detected 1767 unique miRNAs, of which 229 were retained for the primary analysis after abundance filtering. Age- and body mass index (BMI)-adjusted differential-expression analyses identified 50 miRNAs in males with HFpEF and 59 miRNAs in males with HFrEF, whereas no miRNAs reached statistical significance in females with either HF phenotype. Importantly, HF-associated log2 fold changes in males and females were positively correlated for both HF phenotypes, although the absolute magnitudes of change were greater in males than in females, indicating partly shared directions of change between sexes. Age- and BMI-adjusted permutational multivariate analysis of variance revealed more pronounced phenotype-associated differences in global miRNA profiles in males than in females; these findings were consistent across alternative abundance filters.

**Conclusion:**

Sex-stratified analyses of circulating miRNAs in HFpEF and HFrEF showed that HF-associated expression changes were consistently more pronounced in males than in females. These findings highlight the importance of sex stratification when interpreting circulating miRNA alterations in HF.

**Trial registration number:**

UMIN000052673 (UMIN Clinical Trials Registry; observational study)

## Graphical Abstract

**Graphical Abstract:**
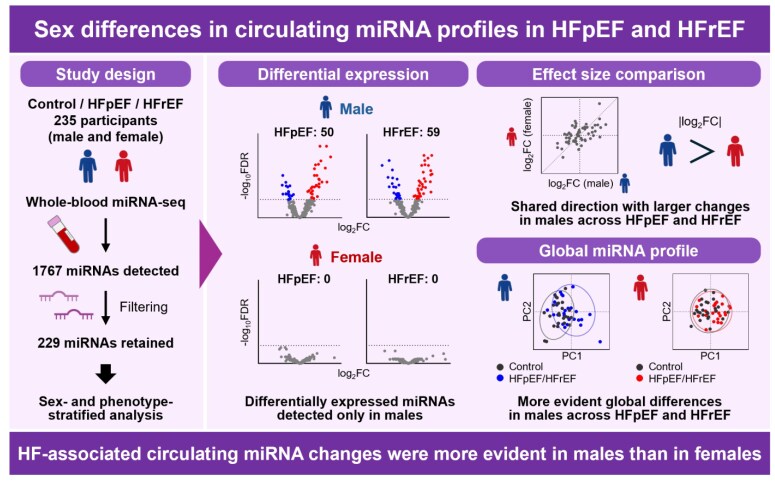

## Introduction

Heart failure (HF) affects > 64 million people worldwide, imposing global socio-economic burden.^1^ HF is a heterogeneous syndrome comprising distinct phenotypes, including HF with preserved ejection fraction (HFpEF) and HF with reduced ejection fraction (HFrEF). Clinical presentation, comorbidity burden, and outcomes differ between these phenotypes, and accumulating evidence suggests that sex also influences HF pathophysiology and clinical characteristics.^2–4^ In particular, HFpEF is more prevalent in females, whereas HFrEF is more common in males, indicating that sex-stratified evaluation may be crucial when investigating HF-associated biological signals.^3,4^

Circulating microRNAs (miRNAs) have emerged as promising biomarkers of cardiovascular disease as they can be detected non-invasively and may reflect pathophysiological processes.^5,6^ Several studies have reported associations between circulating miRNAs and HF-related traits, including cardiac remodelling, inflammation, vascular dysfunction, and metabolic stress.^5–7^ However, many previous studies have analysed males and females together, making it difficult to determine whether HF-associated miRNA changes are comparable between sexes or differ in magnitude and overall profile.^8,9^

In the present study, we performed unbiased sequencing of circulating miRNAs in controls and in patients with HFpEF or HFrEF, with separate analyses in males and females. Using sex-stratified models, we aimed to determine whether HF-associated changes in circulating miRNAs differ between sexes at the individual miRNA level and the global expression profile level.

## Methods

### Study design and participants

We conducted a multicentre, cross-sectional observational study with prospective enrolment at six medical institutions in Japan (UMIN000052673). Adults aged 60–89 years who provided written informed consent were enrolled and classified into three groups: Controls, defined as individuals with no history of HF and at least one cardiovascular risk factor; HFpEF, defined as patients receiving treatment for HF with left ventricular ejection fraction (LVEF) ≥ 45%; and HFrEF, defined as patients receiving treatment for HF with LVEF < 45%. The study protocol was approved by the institutional review boards of all participating centres (approval No. 2023060NI) and adhered to the Declaration of Helsinki. The details of study design and participants are provided in Supplementary Methods and Supplementary material online, *Table S1*.

### miRNA sequencing and analysis

Total RNA, including small RNAs, was extracted from whole-blood samples. miRNA sequencing libraries were prepared and sequenced on an Illumina NextSeq 2000 platform. Adapter-trimmed reads were processed using miRge3.0 and aligned to miRBase v22 to generate raw miRNA counts.^10,11^

For the primary analysis, miRNAs were retained if they had ≥ 10 raw counts in ≥ 50% of samples within at least one of the six sex-by-phenotype subgroups (main filter). To assess the robustness of the findings to the abundance threshold, two additional filters were examined in sensitivity analyses: an intermediate filter (≥5 raw counts in ≥ 30% of samples within at least one of the six sex-by-phenotype subgroups) and a permissive filter (≥3 raw counts in ≥ 10% of samples within at least one of the six sex-by-phenotype subgroups).

Filtered miRNA datasets were subsequently used for age- and body mass index (BMI)-adjusted differential-expression analyses using DESeq2,^12^ between-sex comparisons of log2 fold changes derived from the adjusted models, and age- and BMI-adjusted permutational multivariate analysis of variance (PERMANOVA). The details of miRNA sequencing and analyses are described in Supplementary Methods.

### Statistical analysis

All statistical analyses were performed in R (v4.4.2). Baseline characteristics were compared using Student’s *t*-test or Mann–Whitney U test for two-group comparisons, and analysis of variance (ANOVA) or Kruskal–Wallis test for three-group comparisons, as appropriate. For categorical variables, χ^2^ test or Fisher’s exact test was applied. Between-sex correlations of log2 fold changes were assessed using Spearman correlation coefficients, and differences in absolute log2 fold changes between males and females were evaluated using paired Wilcoxon signed-rank tests. Statistical significance was defined as FDR < 0.05 for differential-expression analyses and two-sided *P* < 0.05 for all other analyses.

## Results

### Patient characteristics

**Figure 1:**
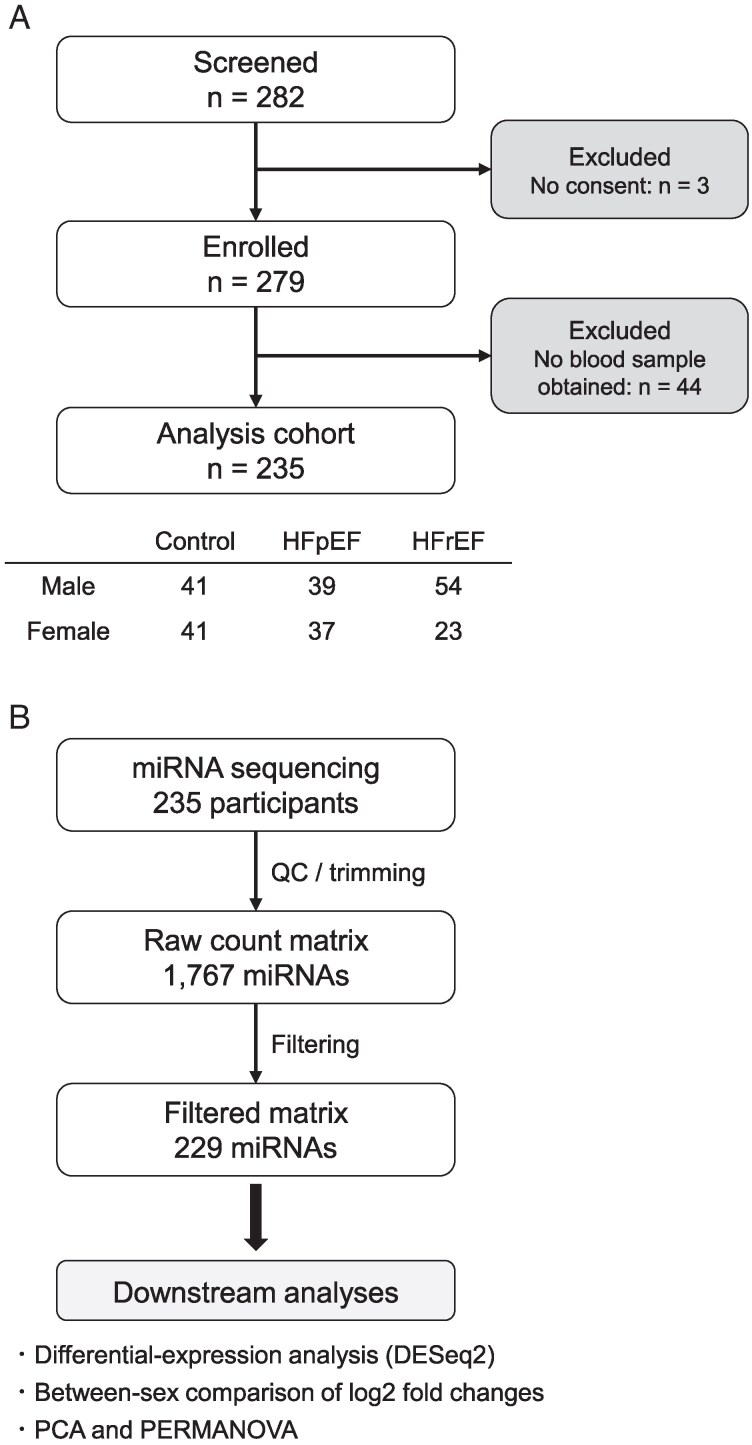
Study cohort and analytic pipeline. (*A*) Study flow diagram showing participant selection and sex distribution across the control, HFpEF, and HFrEF groups. (*B*) Analytic workflow for whole-blood miRNA sequencing. After sequencing and generation of raw miRNA counts, miRNAs were filtered using the main filter (≥10 raw counts in ≥ 50% of samples within at least one of the six sex-by-phenotype subgroups). Primary analyses comprised sex-stratified differential-expression analyses, between-sex comparison of log2 fold changes, and principal component analysis (PCA) with permutational multivariate analysis of variance (PERMANOVA).

The study enrolled 235 participants: 76 with HFpEF (39 males and 37 females), 77 with HFrEF (54 males and 23 females), and 82 controls (41 males and 41 females) (*Figure 1A*). Baseline characteristics stratified by sex and HF phenotype are summarized in *Table 1*, and detailed statistical comparisons for all pairwise group analyses are provided in Supplementary material online, *Table S2*.

**Table 1:** Baseline characteristics

	Control		HFpEF		HFrEF		
	Male (*n* = 41)	Female (*n* = 41)	Male (*n* = 39)	Female (*n* = 37)	Male (*n* = 54)	Female (*n* = 23)	*P*-value
Clinical parameters							
Age (years)	74.0 [67.0–79.0]	73.0 [69.0–79.0]	79.0 [72.0–82.0]	81.0 [76.0–85.0]	72.5 [63.0–77.8]	75.0 [66.5–82.5]	<0.001
BMI (kg/m^2^)	24.4 [21.8–26.1]	24.2 [21.8–26.5]	22.9 [20.3–25.5]	21.2 [18.9–24.7]	23.4 [21.2–25.4]	20.9 [18.8–24.5]*	0.003
SBP (mmHg)	127.0 [115.0–135.0]	129.5 [124.5–140.2]	121.0 [111.0–131.0]	115.0 [110.0–132.0]	103.0 [92.0–119.0]	115.0 [92.0–123.0]	<0.001
DBP (mmHg)	75.0 [69.0–80.0]	73.0 [67.5–79.0]	70.0 [61.0–79.0]	65.0 [56.0–73.0]	62.0 [55.0–68.0]	61.0 [54.0–70.5]	<0.001
Heart rate (b.p.m.)	75.0 [60.8–82.2]	70.0 [66.0–76.0]	72.0 [64.5–80.0]	70.0 [64.0–76.0]	70.0 [62.0–75.0]	73.0 [69.2–75.0]	NS
NYHA class							
I	−	−	3 (7.7%)	2 (5.4%)	3 (5.6%)	2 (8.7%)	
II	−	−	29 (74.4%)	32 (86.5%)	38 (70.4%)	13 (56.5%)	
III	−	−	7 (17.9%)	3 (8.1%)	13 (24.1%)	8 (34.8%)	
IV	−	−	0 (0.0%)	0 (0.0%)	0 (0.0%)	0 (0.0%)	
H_2_FPEF score	2 [2–3]	2 [2–3]	6 [3–6]	6 [3–7]	3 [3–6]	4 [3–6]	<0.001
Medical history							
HF Hospitalization	−	−	23 (59.0%)	25 (67.6%)	39 (72.2%)	19 (82.6%)	−
Hypertension	36 (87.8%)	39 (95.1%)	28 (71.8%)	29 (78.4%)	35 (64.8%)	9 (39.1%)*	<0.001
Dyslipidaemia	29 (70.7%)	32 (78.0%)	14 (35.9%)	23 (62.2%)*	30 (55.6%)	11 (47.8%)	0.002
Diabetes mellitus	13 (31.7%)	9 (22.0%)	13 (33.3%)	12 (32.4%)	22 (40.7%)	4 (17.4%)*	NS
Atrial fibrillation	6 (14.6%)	3 (7.3%)	26 (66.7%)	23 (62.2%)	23 (42.6%)	11 (47.8%)	<0.001
Ischaemic heart disease	15 (36.6%)	2 (4.9%)*	12 (30.8%)	12 (32.4%)	18 (33.3%)	3 (13.0%)	NS
Previous Stroke	1 (2.4%)	0 (0.0%)	3 (7.7%)	6 (16.2%)	3 (5.6%)	1 (4.3%)	0.017
Treatments							
CCB	26 (63.4%)	26 (63.4%)	12 (30.8%)	13 (35.1%)	6 (11.1%)	2 (8.7%)	<0.001
BB	10 (24.4%)	12 (29.3%)	31 (79.5%)	29 (78.4%)	52 (96.3%)	22 (95.7%)	<0.001
MRA	2 (4.9%)	0 (0.0%)	16 (41.0%)	19 (51.4%)	42 (77.8%)	22 (95.7%)	<0.001
ACE-I/ARB	27 (65.9%)	29 (70.7%)	16 (41.0%)	7 (18.9%)*	20 (37.0%)	10 (43.5%)	<0.001
ARNI	4 (9.8%)	5 (12.2%)	16 (41.0%)	19 (51.4%)	30 (55.6%)	12 (52.2%)	<0.001
SGLT2-I	5 (12.2%)	3 (7.3%)	31 (79.5%)	25 (67.6%)	43 (79.6%)	19 (82.6%)	<0.001
sGC	0 (0.0%)	0 (0.0%)	1 (2.6%)	0 (0.0%)	7 (13.0%)	4 (17.4%)	<0.001
Diuretics	2 (4.9%)	0 (0.0%)	17 (43.6%)	23 (62.2%)	36 (66.7%)	15 (65.2%)	<0.001
Statin	23 (56.1%)	29 (70.7%)	17 (43.6%)	22 (59.5%)	29 (53.7%)	13 (56.5%)	NS
Insulin	2 (4.9%)	0 (0.0%)	0 (0.0%)	0 (0.0%)	1 (1.9%)	1 (4.3%)	NS
OHA	13 (31.7%)	4 (9.8%)*	8 (20.5%)	5 (13.5%)	7 (13.0%)	3 (13.0%)	NS
PCI	19 (46.3%)	1 (2.4%)*	7 (17.9%)	11 (29.7%)	16 (29.6%)	2 (8.7%)*	NS
CABG	0 (0.0%)	0 (0.0%)	3 (7.7%)	1 (2.7%)	3 (5.6%)	1 (4.3%)	NS
Laboratory parameters							
BNP (pg/mL)	25.6 [13.5–43.3]	28.7 [19.6–55.6]	243.0 [131.0–393.0]	232.0 [135.0–406.0]	238.5 [119.0–406.8]	190.0 [102.2–551.0]	<0.001
eGFR (mL/min/1.73m^2^)	61.3 [50.8–72.5]	63.8 [54.9–74.9]	46.2 [35.3–62.6]	37.1 [31.7–49.2]*	46.7 [37.8–55.4]	46.1 [37.0–54.8]	<0.001
Hb (g/dL)	14.4 [13.2–15.3]	12.8 [12.4–13.6]*	14.1 [12.5–15.2]	12.4 [11.4–13.7]*	14.5 [12.9–15.3]	13.9 [12.5–14.9]	0.018
HbA1c (%)	6.1 [5.8–6.8]	6.0 [5.8–6.3]	6.2 [5.8–6.4]	6.2 [6.0–6.6]	6.2 [5.8–7.0]	6.0 [5.6–6.3]	NS
LDL-C (mg/dL)	88.0 [70.0–107.0]	115.0 [98.0–132.0]*	77.0 [68.2–106.0]	79.0 [69.2–105.2]	77.0 [59.0–97.5]	96.5 [80.2–127.8]*	<0.001
HDL-C (mg/dL)	64.0 [46.7–72.1]	68.0 [55.6–83.6]	61.9 [52.5–72.8]	55.5 [49.2–69.6]	48.2 [40.0–62.5]	66.0 [49.1–85.3]*	0.007
TG (mg/dL)	108.0 [69.0–134.2]	111.0 [76.0–144.0]	85.5 [63.5–119.8]	86.5 [73.2–141.2]	136.5 [85.5–180.2]	108.0 [92.2–161.8]	0.012
Echocardiographic parameters							
LVEF (%)	66.0 [62.8–71.0]	71.0 [66.0–73.0]	57.0 [52.2–63.5]	64.0 [60.7–66.8]*	31.5 [21.2–40.8]	33.0 [25.4–41.0]	<0.001
E/A	0.8 [0.6–0.9]	0.8 [0.7–0.9]	1.0 [0.7–2.8]	1.0 [0.7–4.2]	0.7 [0.6–0.8]	2.0 [0.8–2.9]*	0.009
*E*/*e*´	9.3 [7.4–11.2]	9.9 [8.6–11.7]	10.4 [9.0–17.8]	12.7 [10.1–19.5]	11.8 [8.5–15.0]	14.7 [10.3–28.4]*	<0.001
LVDd (mm)	44.5 [41.8–48.2]	42.0 [39.5–44.5]*	50.0 [46.0–52.0]	44.0 [37.0–47.0]*	59.0 [55.0–67.0]	53.0 [50.5–58.5]*	<0.001
LVDs (mm)	28.0 [25.0–31.0]	26.0 [23.5–28.0]*	34.0 [30.0–36.0]	26.0 [25.0–31.0]*	50.0 [45.0–61.0]	47.0 [40.0–50.0]*	<0.001
IVSd (mm)	9.0 [8.0–10.0]	10.0 [8.0–10.0]	10.0 [9.0–11.0]	9.0 [9.0–10.0]	8.0 [7.0–9.0]	7.0 [6.0–9.8]	<0.001
PWd (mm)	9.0 [8.8–10.0]	9.0 [8.0–10.0]	10.0 [9.0–11.0]	9.0 [8.0–10.0]*	8.0 [8.0–10.0]	8.7 [7.0–9.0]	0.002
LAD (mm)	36.0 [32.5–40.5]	37.0 [32.0–40.5]	46.0 [41.0–51.5]	43.0 [36.0–48.0]*	42.0 [36.0–49.0]	41.0 [31.8–44.5]	<0.001
LAVI (mL/m^2^)	26.0 [21.5–31.5]	27.0 [22.0–37.0]	49.6 [38.0–85.0]	41.8 [30.5–60.2]	48.5 [35.8–69.0]	40.6 [24.7–49.0]	<0.001

Continuous variables are reported as median [inter-quartile range], and categorical variables as *n* (%). *P*-values represent overall comparisons among the three phenotypes (Control, HFpEF, and HFrEF): normally distributed variables by one-way ANOVA, non-normal variables by Kruskal–Wallis test, and categorical variables by χ^2^ test or Fisher’s exact test, as appropriate. *P < 0.05 vs. males within the same phenotype. ACE-I, angiotensin-converting enzyme inhibitor; ARB, angiotensin II receptor blocker; ARNI, angiotensin receptor–neprilysin inhibitor; BB, beta-blocker; BMI, body mass index; BNP, B-type natriuretic peptide; CABG, coronary artery bypass grafting; CCB, calcium channel blocker; DBP, diastolic blood pressure; E/A, early-to-late diastolic mitral inflow velocity ratio; E/e′, early diastolic mitral inflow velocity to mitral annular velocity ratio; eGFR, estimated glomerular filtration rate; Hb, haemoglobin; HbA1c, haemoglobin A1c; HDL-C, high-density lipoprotein cholesterol; HF, heart failure; HFpEF, heart failure with preserved ejection fraction; HFrEF, heart failure with reduced ejection fraction; IVSd, interventricular septal thickness at diastole; LAD, left atrial diameter; LAVI, left atrial volume index; LDL-C, low-density lipoprotein cholesterol; LVDd, left ventricular end-diastolic diameter; LVDs, left ventricular end-systolic diameter; LVEF, left ventricular ejection fraction; MRA, mineralocorticoid receptor antagonist; NS, not significant; NYHA, New York Heart Association; OHA, oral hypoglycaemic agent; PCI, percutaneous coronary intervention; PWd, posterior wall thickness at diastole; SBP, systolic blood pressure; sGC, soluble guanylate cyclase; SGLT2-I, sodium–glucose cotransporter 2 inhibitor; TG, triglycerides.

The overall median age was 76 years [inter-quartile range (IQR) 67–82]. Patients with HFpEF were significantly older than those with HFrEF or controls, with female HFpEF patients having the highest median age (81 years, IQR 76–85). BMI was lower in HFpEF patients compared with controls, without a sex difference, whereas in HFrEF, BMI was lower in females than in males (20.9 vs. 23.4 kg/m^2^). H_2_FPEF score was consistent with the diagnosis, showing a median of 6 in HFpEF in both sexes. Plasma BNP concentrations were comparable between sexes in both HFpEF (median 243 pg/mL in males, 232 pg/mL in females) and HFrEF (239 pg/mL in males, 190 pg/mL in females).

### Sex-stratified differential-expression analyses

**Figure 2:**
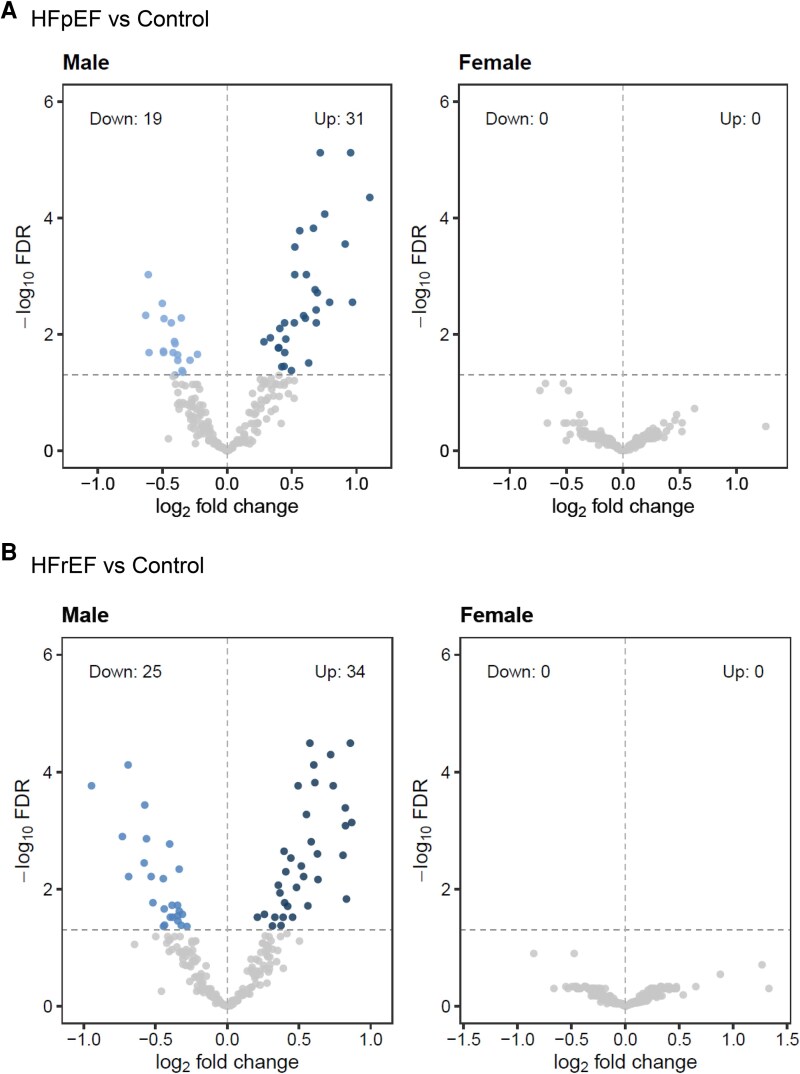
Sex-stratified volcano plots of circulating miRNA expression changes in HFpEF and HFrEF. (*A*, *B*) Volcano plots showing age- and BMI-adjusted differential-expression results for sex-stratified comparisons of HFpEF vs. Control (*A*) and HFrEF vs. Control (*B*). The x-axis indicates log2 fold change for the HF phenotype relative to Control, and the y-axis indicates −log10(FDR). Points passing FDR < 0.05 are highlighted. In males, 50 miRNAs were significantly altered in HFpEF (31 up-regulated and 19 down-regulated), and 59 miRNAs were significantly altered in HFrEF (34 up-regulated and 25 down-regulated). No miRNAs met the FDR threshold in females for either comparison.

Whole-blood miRNA sequencing detected 1767 unique miRNAs across the cohort, of which 229 were retained for the primary analyses using the main filter (≥10 raw counts in ≥ 50% of samples within at least one of the six sex-by-phenotype subgroups) (*Figure 1B*). Using DESeq2 models adjusted for age and BMI, 50 miRNAs were differentially expressed in males with HFpEF compared with male controls, whereas no miRNAs met the FDR threshold (<0.05) in females with HFpEF (*Figure 2A*). Similarly, 59 miRNAs were differentially expressed in males with HFrEF compared with male controls, whereas no significant miRNAs were identified in females with HFrEF (*Figure 2B*). The details of DESeq2 results are provided in Supplementary material online, *Table S3*.

### Between-sex comparison of HF-associated changes in miRNA profiles

**Figure 3:**
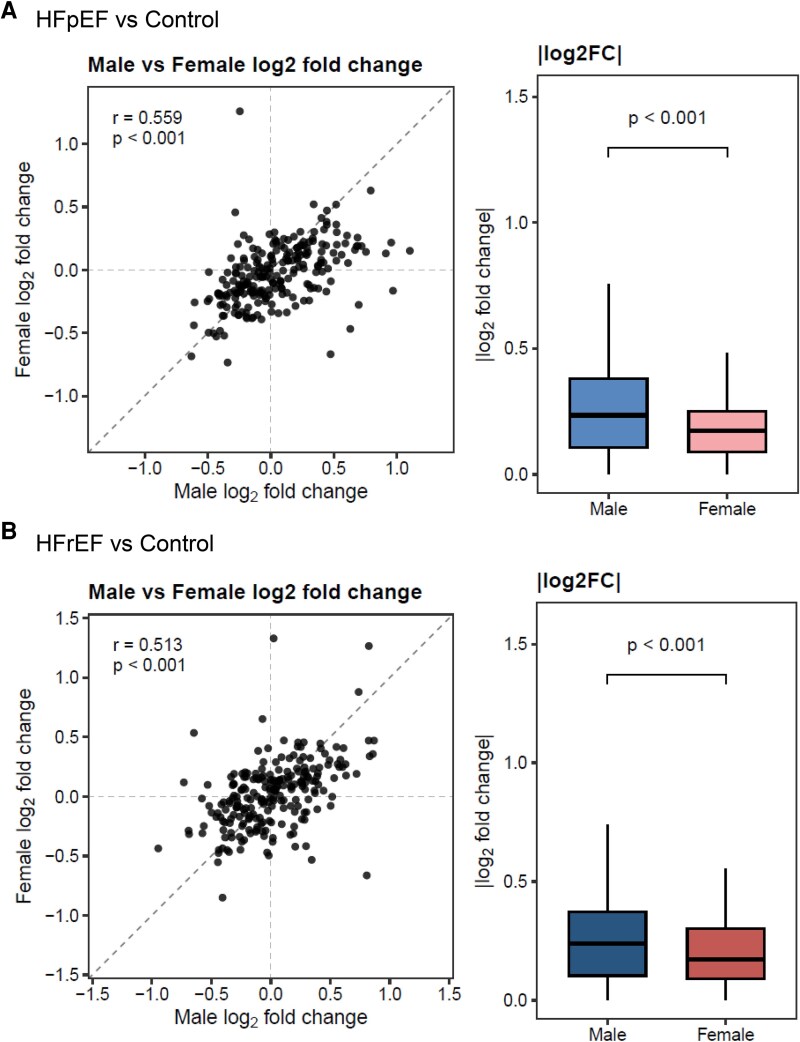
Between-sex comparison of HF-associated miRNA changes. (*A*) HFpEF vs. Control. (*B*) HFrEF vs. Control. In each panel, the left scatter plot compares age- and BMI-adjusted log2 fold changes relative to Control in males (x-axis) and females (y-axis) for the 229 filtered miRNAs, with each point representing one miRNA. The corresponding Spearman correlation coefficients were 0.559 for HFpEF vs. Control and 0.513 for HFrEF vs. Control (both *P* < 0.001). The right box plot compares absolute age- and BMI-adjusted log2 fold changes between males and females across the same set of analysed miRNAs. Boxes indicate the inter-quartile range (IQR), with the median shown as the centre line; whiskers indicate the range within 1.5 × IQR. Paired Wilcoxon signed-rank tests were used.

Although no individual miRNAs reached statistical significance in females, male and female log2 fold changes were positively correlated across the 229 analysed miRNAs in both HF phenotypes, with Spearman correlation coefficients of 0.559 for HFpEF vs. Control and 0.513 for HFrEF vs. Control (both *P* < 0.001) (*Figure 3*, left panels).

The magnitude of HF-associated expression changes, however, was greater in males than in females. Across all analysed miRNAs, absolute log2 fold changes for matched miRNAs were significantly larger in males than in females for both HFpEF vs. Control and HFrEF vs. Control (both *P* < 0.001) (*Figure 3*, right panels). These findings suggest that the direction of HF-associated miRNA changes was partly shared between sexes, whereas the magnitude of those changes was attenuated in females.

### Sex-stratified changes in global miRNA profiles

**Figure 4:**
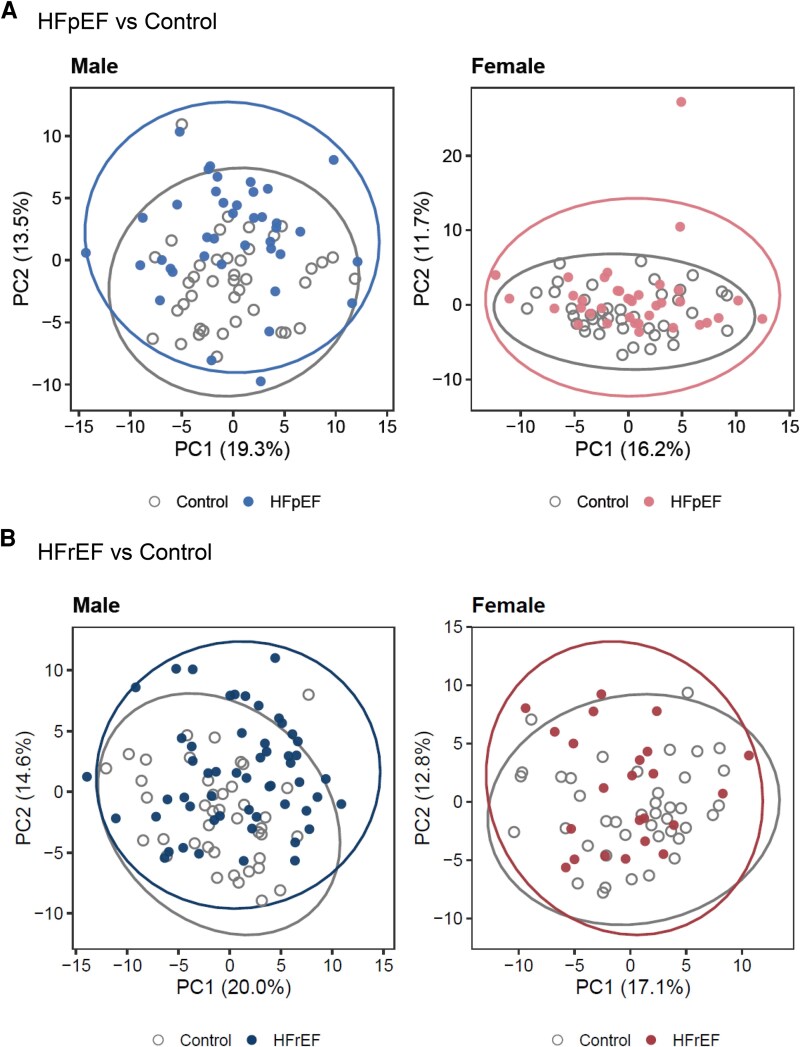
Principal component analysis of circulating miRNA profiles. (*A*, *B*) Principal component analysis (PCA) plots based on variance-stabilized expression values from the 229 filtered miRNAs for HFpEF vs. Control (*A*) and HFrEF vs. Control (*B*). Males and females are shown separately. Control samples are shown as open circles and HF samples as filled circles. Ellipses indicate 95% normal-data regions for each group.

Principal component analysis (PCA) of the 229 filtered miRNAs showed a tendency towards greater separation between HF and control samples in males than in females for both HFpEF and HFrEF comparisons (*Figure 4*). To evaluate these global differences, sex-stratified PERMANOVA with adjustment for age and BMI was performed. In males, phenotype remained significantly associated with global miRNA profiles for both HFpEF vs. Control and HFrEF vs. Control (both *P* < 0.001). In contrast, phenotype was not statistically significant in females for HFpEF vs. Control or HFrEF vs. Control (see Supplementary material online, *Table S4*).

### Sensitivity analyses across alternative abundance filters

To assess the robustness of the findings to the abundance-filtering strategy, the analyses were repeated using two additional filters. The intermediate filter (≥5 raw counts in ≥ 30% of samples within at least one of the six sex-by-phenotype subgroups) and the permissive filter (≥3 raw counts in ≥ 10% of samples within at least one of the six sex-by-phenotype subgroups) retained 328 and 558 miRNAs, respectively (see Supplementary material online, *Figure S1*).

The overall pattern was broadly preserved across filters: no individual miRNA reached FDR < 0.05 in females for either HFpEF or HFrEF under any filter, whereas males consistently showed multiple differentially expressed miRNAs (see Supplementary material online, *Figure S2A*). Male HFpEF yielded 50, 54, and 54 significant miRNAs under the main, intermediate, and permissive filters, respectively, whereas male HFrEF yielded 59, 59, and 58 significant miRNAs. Between-sex correlations of log2 fold changes remained positive across filters, and the larger absolute log2 fold changes in males were preserved for HFpEF under any filter (all *P* < 0.001), although the sex difference was attenuated for HFrEF under the permissive filter (*P* = 0.081) (see Supplementary material online, *Figures S2B* and *S2C*). PERMANOVA results showed a similar overall pattern across filters, with phenotype remaining significantly associated with global miRNA profiles in males but not in females (see Supplementary material online, *Figure S3*).

## Discussion

In this study, sex-stratified analyses of circulating miRNAs revealed marked differences in the magnitude of HF-associated expression changes between males and females. Differentially expressed miRNAs were identified in males in both HFpEF and HFrEF comparisons, whereas no miRNAs reached statistical significance in females. At the same time, HF-associated log2 fold changes were directionally concordant between sexes, but their absolute magnitudes were consistently greater in males. PERMANOVA-based analyses of global miRNA profiles similarly indicated more evident phenotype-associated differences in males than in females. Taken together, these findings suggest that HF-associated circulating miRNA perturbations were more pronounced in males, extending prior studies of circulating miRNA signatures in HFpEF and HFrEF that did not explicitly address sex differences.^8,9^

Although robust differentially expressed miRNAs were not detected in females, HF-associated log2 fold changes were positively correlated between sexes, indicating partly shared directions of change. This suggests that the observed sex difference is better interpreted as a difference in the magnitude of miRNA perturbation rather than a complete absence of HF-associated changes in females. Although the absence of statistically significant miRNAs in females may partly reflect smaller sample size and reduced power, the effect-size analyses indicate that this pattern is not explained by significance thresholds alone. Thus, females may still exhibit HF-associated circulating miRNA changes, but in the present cohort these changes were smaller in magnitude than those observed in males.

Several factors may underlie these sex-related differences in circulating miRNA responses, including differences in disease biology, comorbidity structure, hormonal environment, body composition, and the systemic response to HF-related stress. At the same time, our study was not designed to establish causal mechanisms, and the observed pattern should not be interpreted as evidence of inherently sex-specific molecular pathways. Rather, the present data indicate that sex-stratified analyses can reveal patterns of HF-associated circulating miRNA changes that may be obscured in pooled analyses.

This study has several limitations. First, the cohort size was modest, particularly for females with HFrEF, which may have reduced power to detect weaker female-associated signals. Second, the analyses were cross-sectional and therefore do not address temporal changes or causal relationships. Third, although we adjusted for age and BMI, residual confounding by other clinical factors cannot be excluded. Fourth, because this study was conducted exclusively in Japan and the sex-stratified findings were not externally validated in an independent cohort, their generalizability to other populations remains uncertain.

## Conclusions

Sex-stratified analyses of circulating miRNAs in HFpEF and HFrEF revealed a consistent pattern of more pronounced HF-associated expression changes in males than in females. These findings highlight the importance of sex stratification when interpreting circulating miRNA alterations in HF.

## Supplementary Material

oeag073_Supplementary_Data

## Data Availability

The raw sequencing data supporting our findings are available in the NCBI Sequence Read Archive (BioProject PRJNA1293890).
