# Supplementary material for: Sex differences in circulating microRNA profiles in heart failure with preserved and reduced ejection fraction
Source: Eur Heart J Open. 2026 May 13;6(3):oeag073. doi: 10.1093/ehjopen/oeag073 (PMC13252451; doi:10.1093/ehjopen/oeag073)
Supplement: oeag073_Supplementary_Data [file oeag073_supplementary_data.docx]

**SUPPLEMENTARY MATERIALS**

**Sex Differences in Circulating MicroRNA Profiles in Heart Failure with Preserved and Reduced Ejection Fraction**

Genri Numata^1,2*^, Shun Nakamura^2,3*^, Hiroyuki Tokiwa^2^, Akashi Taguchi^1^, Masayuki Toyoda^2^, Takashi Kohno^4^, Hisataka Maki^5^, Yasuyuki Shiraishi^6^, Shinsuke Takeuchi^4^, Ayumi Goda^4^, Kyohei Daigo^6^, Natsuko Yamamoto^2,5^, Yusuke Adachi^2^, Kosuke Yabe^7^, Takao Kato^8^, Yukio Hiroi^9^, Norihiro Kato^10^, Takuya Kawahara^11^, Kazutaka Ueda^2,12^, Eisuke Amiya^2^, Masaru Hatano^2,13^, Hideo Fujita^5^, Masaki Ieda^6^, Tetsuo Sasano^3^, Norihiko Takeda^2^, Takahide Kohro^14^, Yasushi Hirota^15^, Issei Komuro^12,16^, Youichiro Wada^1^, Eiki Takimoto^2,10,17^

**Affiliations**
^1^ Isotope Science Center, The University of Tokyo, Tokyo, Japan.

^2^ Department of Cardiovascular Medicine, The University of Tokyo Hospital, Tokyo, Japan.

^3^ Department of Cardiovascular Medicine, Institute of Science Tokyo, Tokyo, Japan.

^4^ Department of Cardiovascular Medicine, Kyorin University School of Medicine, Tokyo, Japan.

^5^ Division of Cardiovascular Medicine, Saitama Medical Center, Jichi Medical University, Saitama, Japan.

^6^ Department of Cardiology, Keio University School of Medicine, Tokyo, Japan.

^7^ Horikiri Central Hospital, Tokyo, Japan.

^8^ Department of Cardiovascular Biology and Medicine, Juntendo University Graduate School of Medicine, Tokyo, Japan.

^9^ Department of Cardiology, National Center for Global Health and Medicine, Japan Institute for Health Security, Tokyo, Japan.

^10^ Medical Genomics Center, National Institute of Global Health and Medicine, Japan Institute for Health Security, Tokyo, Japan.

^11^ Clinical Research Promotion Center, The University of Tokyo Hospital, Tokyo, Japan.

^12^ International University of Health and Welfare, Tokyo, Japan.

^13^ Advanced Medical Center for Heart Failure, The University of Tokyo Hospital, Tokyo, Japan.

^14^ Department of Clinical Informatics, Jichi Medical University School of Medicine, Tochigi, Japan.

^15^ Department of Obstetrics and Gynecology, Graduate School of Medicine, The University of Tokyo, Tokyo, Japan.

^16^ Department of Frontier Cardiovascular Science, Graduate School of Medicine, The University of Tokyo, Tokyo, Japan.

^17^ Division of Cardiology, Department of Medicine, The Johns Hopkins Medical Institutions, Baltimore, MD, USA.

*These authors contributed equally to this work.

**Correspondence:**

Eiki Takimoto, MD, PhD

Department of Cardiovascular Medicine, The University of Tokyo Hospital, 7-3-1 Hongo, Bunkyo-ku, Tokyo, 113-8655, Japan.

Division of Cardiology, The Johns Hopkins Medical Institutions, 720 Rutland Ave., Baltimore, MD 21205, USA.

Medical Genomics Center, National Institute of Global Health and Medicine, Japan Institute for Health Security, 1-21-1 Toyama, Shinjuku-ku, Tokyo, 162-8655, Japan.

Email: eikitakimoto@gmail.com or etakimo1@jhmi.edu or takimoto.e@jihs.go.jp

Phone: +81-3-3815-5411

Fax: +81-3-5800-9780

**Table of Contents:**

Supplementary Methods

Supplementary Figures S1-S3

Supplementary Tables S1-S4

**Supplementary Methods**

**Study design and participants**

We undertook a multicenter, cross-sectional observational study with prospective enrollment at six medical institutions in Japan (trial registration: UMIN000052673): The University of Tokyo Hospital, Kyorin University Hospital, Jichi Medical University Saitama Medical Center, Keio University Hospital, Juntendo University Hospital, and Horikiri Central Hospital.

Adults aged 60–89 years were screened across these centers between November 2023 and April 2025, and eligible individuals who provided informed consent were enrolled. For the present microRNA (miRNA) analysis, participants were eventually enrolled from five of the six institutions―The University of Tokyo Hospital, Kyorin University Hospital, Jichi Medical University Saitama Medical Center, Keio University Hospital, and Horikiri Central Hospital.

Participants were classified into three groups: a control group, defined as individuals with no history of heart failure (HF) and at least one cardiovascular risk factor (hypertension, diabetes mellitus, atrial fibrillation, or obesity); a HF with preserved ejection fraction (HFpEF) group, comprising patients receiving treatment for HF with left ventricular ejection fraction (LVEF) ≥ 45%; and a HF with reduced ejection fraction (HFrEF) group, comprising patients receiving treatment for HF with LVEF < 45%. Only clinically stable HF patients without a history of HF hospitalization within the past month were included. Other key exclusion criteria—including active malignancy, end-stage renal disease, and specific cardiomyopathies—are detailed in **Supplementary Table S1**. The protocol was approved by the institutional review boards of all participating centers (approval No. 2023060NI) and complied with the Declaration of Helsinki.

**miRNA sequencing**
At the outpatient visit following the provision of informed consent, blood samples were collected for the analyses including miRNA sequencing. Blood aliquots were processed centrally. Total RNA, including small RNA, was extracted from whole blood using the PAXgene Blood miRNA Kit (Qiagen). Indexed libraries were prepared with the QIAseq miRNA Library Kit (96-plex) and pooled for single-end 72-cycle sequencing on an Illumina NextSeq 2000 (P2 XLEAP-SBS chemistry). Adapter-trimmed reads were aligned to miRBase v22 with miRge3.0 and summarized as raw counts.^10,11^ Library preparation and sequencing were carried out in two balanced batches, blinded to phenotype.

**Differential-expression analysis**

Differential-expression analyses were performed separately in males and females for the comparisons of HFpEF vs. Control and HFrEF vs. Control using DESeq2.^12^ In each sex-stratified comparison, age and body mass index (BMI) were included as covariates. Differential expressions were assessed for each HF phenotype relative to the corresponding control group. Multiple testing was controlled using the Benjamini–Hochberg false discovery rate (FDR) method, and miRNAs with FDR < 0.05 were considered differentially expressed.

**Between-sex comparison of differential expression magnitude**

To compare HF-associated miRNA changes between sexes, male and female differential-expression results were matched by miRNA for each phenotype comparison. Concordance in the direction of change between sexes was assessed using Spearman correlation coefficients calculated from age- and BMI-adjusted log2 fold changes across all analyzed miRNAs. To compare the magnitude of HF-associated changes, absolute log2 fold changes were compared between males and females using paired Wilcoxon signed-rank tests across matched miRNAs. These analyses were conducted separately for HFpEF vs. Control and HFrEF vs. Control.

**PCA and PERMANOVA**

To visualize global expression patterns, the filtered count matrix was variance-stabilized before downstream multivariate analyses. Principal component analysis (PCA) was then performed separately for each sex and phenotype comparison.

To evaluate differences in global miRNA expression profiles between phenotypes after accounting for age and BMI, permutational multivariate analysis of variance (PERMANOVA) was performed separately for each sex-stratified comparison using Euclidean distances derived from variance-stabilized expression data. Age and BMI were included as covariates, and the effects of phenotype, age, and BMI were evaluated using 9,999 permutations.

**Supplementary Figures**

**
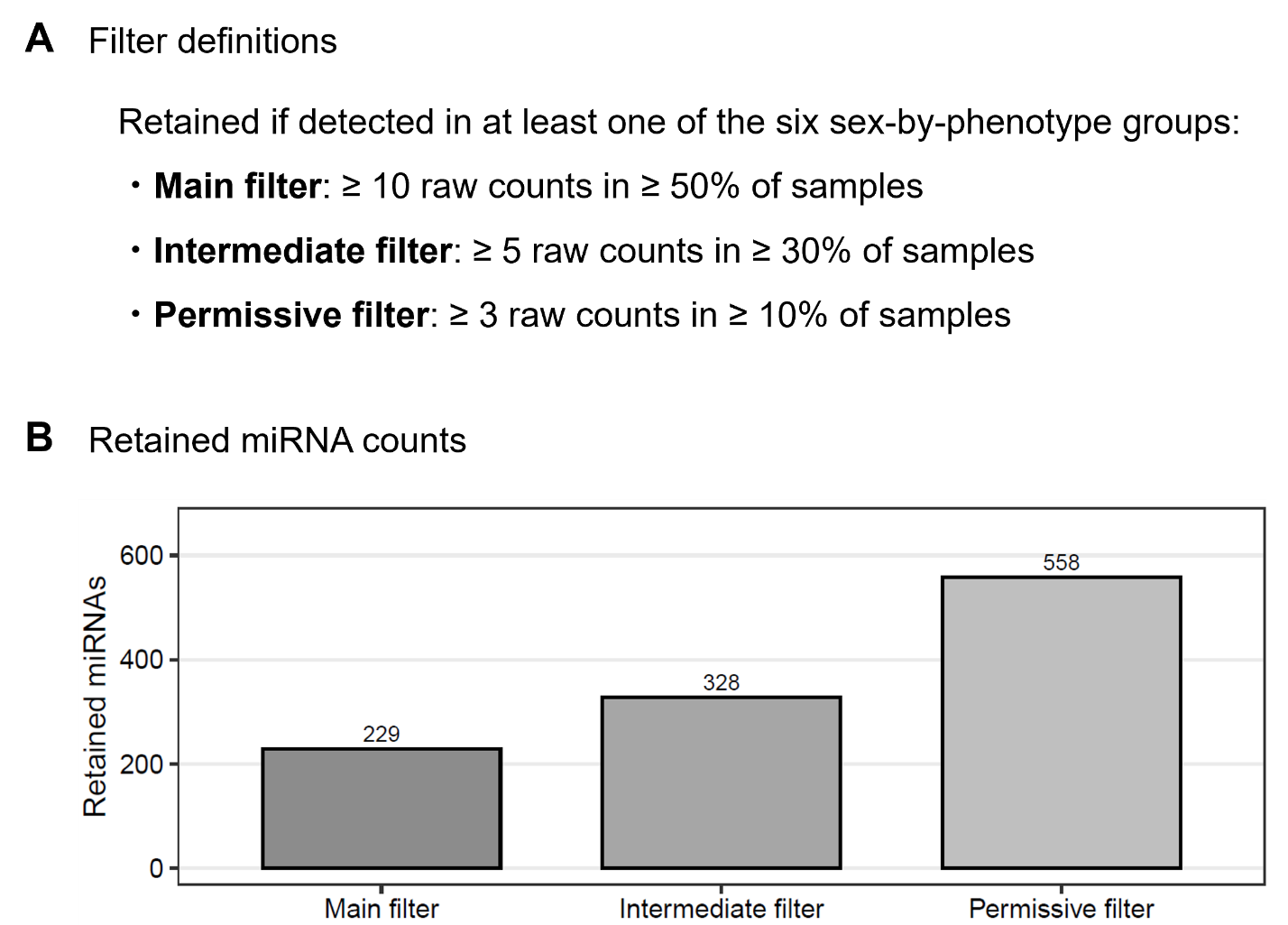
**

**Supplementary Figure S1. Overview of abundance-filter definitions and retained miRNAs.**

**(A)** Definitions of the three abundance filters. miRNAs were retained if they met the following criteria in at least one of the six sex-by-phenotype groups: main filter, ≥ 10 raw counts in ≥ 50% of samples; intermediate filter, ≥ 5 raw counts in ≥ 30% of samples; and permissive filter, ≥ 3 raw counts in ≥ 10% of samples.

**(B)** Number of miRNAs retained under each filter.

**
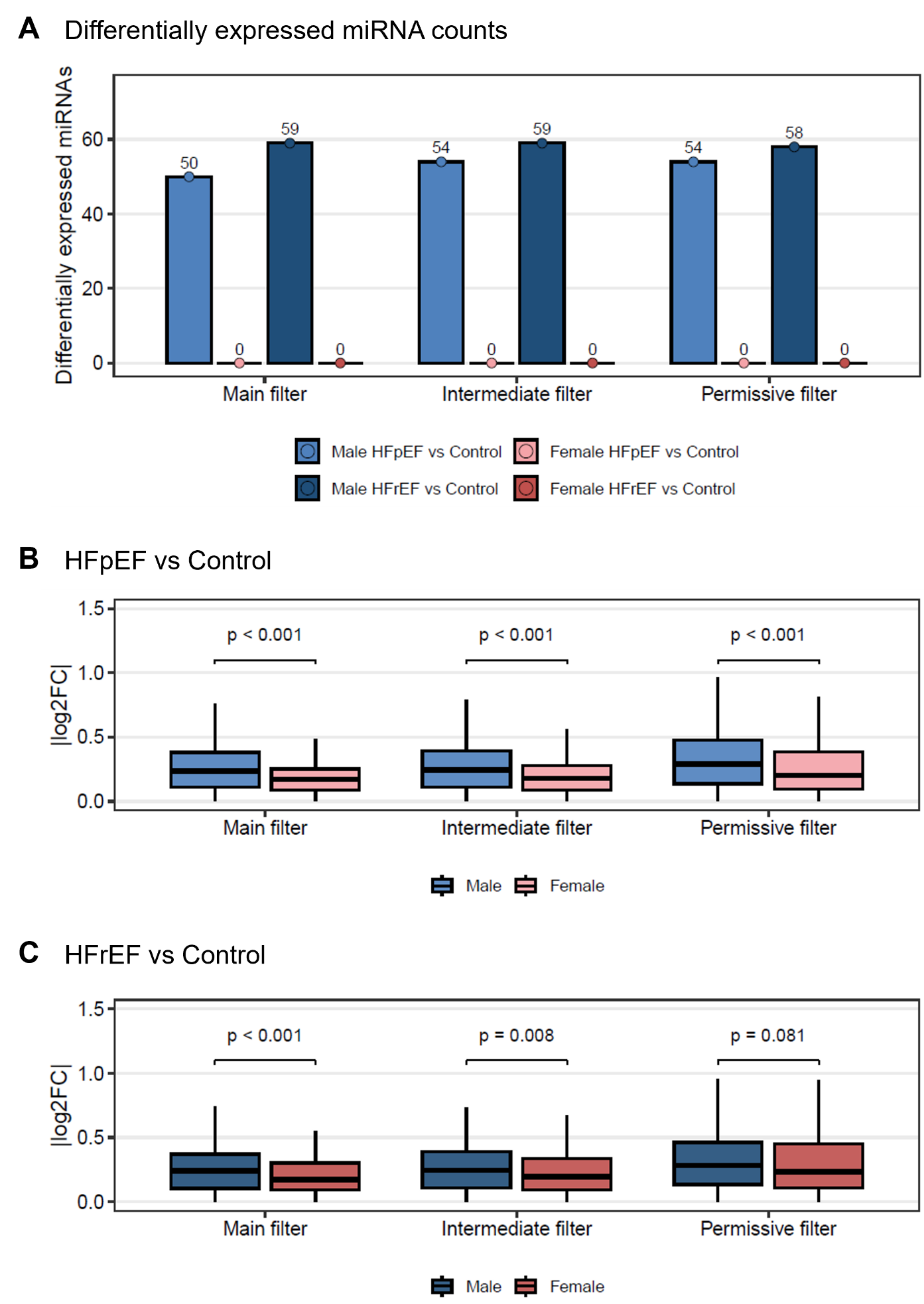
**

**Supplementary Figure S2. Sensitivity analyses of differential-expression counts and effect-size distributions across filter definitions.**

**(A)** Numbers of differentially expressed miRNAs (FDR < 0.05) in each sex-stratified comparison under the main, intermediate, and permissive filters.

**(B, C)** Box plots of absolute age- and BMI-adjusted log2 fold changes in males and females for HFpEF vs. Control **(B)** and HFrEF vs. Control **(C)** across the three filter definitions. The main filter results correspond to those shown in **Figure 3**. Boxes indicate the interquartile range (IQR), with the median shown as the center line; whiskers indicate the range within 1.5 × IQR. Paired Wilcoxon signed-rank tests were used.

**
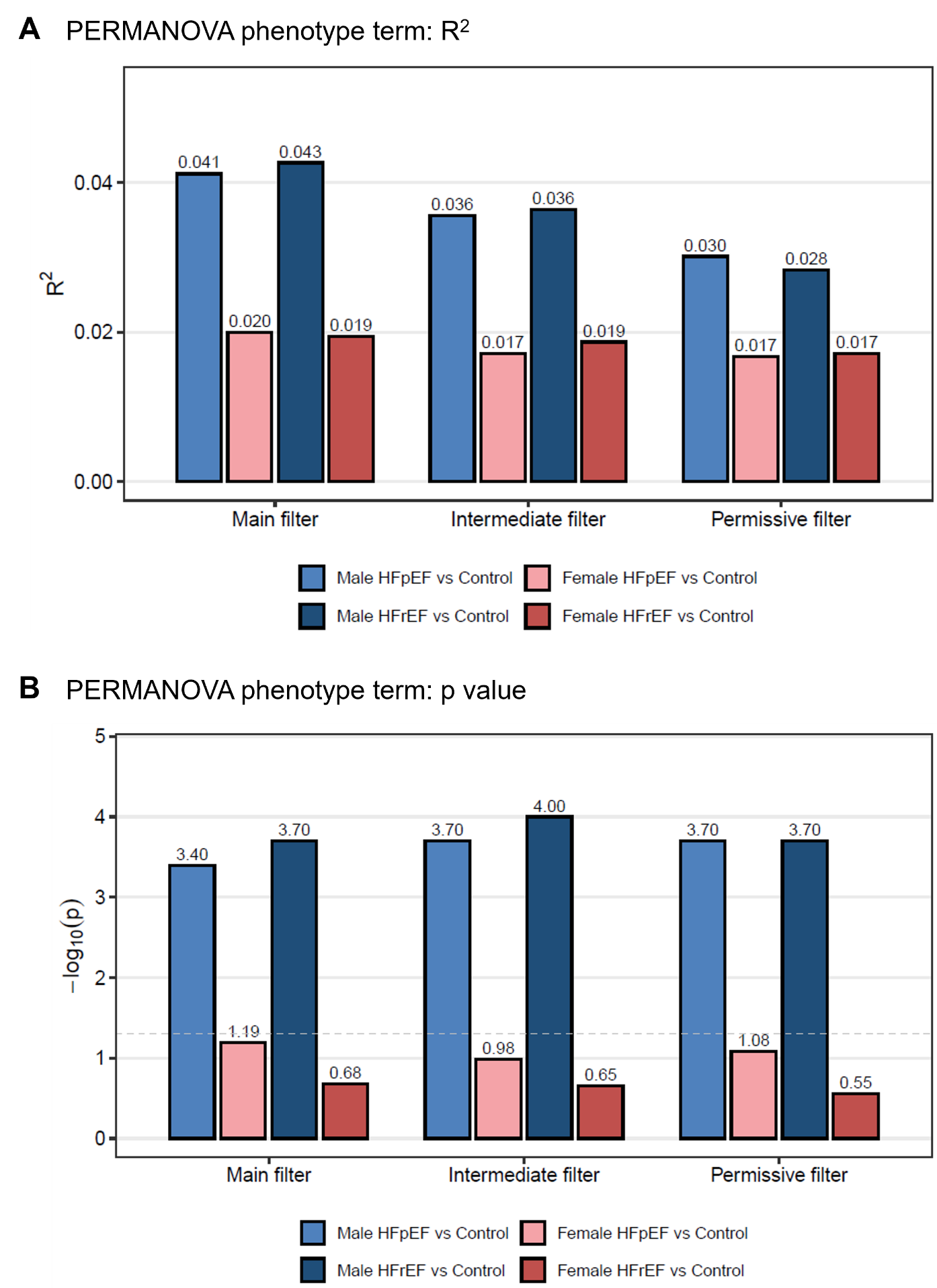
**

**Supplementary Figure S3. Sensitivity analyses of PERMANOVA phenotype effects across filter definitions.
(A, B)** Phenotype-term R² values **(A)** and corresponding P-values shown as −log10(P) **(B)** from sex-stratified PERMANOVA models adjusted for age and BMI for HFpEF vs. Control and HFrEF vs. Control under the main, intermediate, and permissive filters. Across all filter settings, phenotype effects were associated with larger R² values and lower P-values in males than in females. The dashed horizontal line in **(B)** indicates the nominal significance threshold (P = 0.05).

**Supplementary Tables**

| Sex‑hormone replacement therapy for menopausal symptoms or malignant tumors within the past year |
| --- |
| Active infectious disease at the time of sample collection |
| Active malignancy |
| Ongoing immunosuppressive therapy for inflammatory disease |
| Severe renal dysfunction (eGFR < 20 mL/min/1.73 m²) |
| Severe COPD (FEV₁/FVC < 70 % and %FEV₁ < 50 %) |
| Severe hepatic impairment with hepatic encephalopathy |
| History of alcohol or substance abuse |
| Stroke, aortic, or peripheral arterial intervention within the past year |
| Acute coronary syndrome within the past 3 months |
| Decompensated heart failure due to uncontrolled tachyarrhythmia |
| Severe primary valvular heart disease |
| Within three months after valvular intervention |
| Diagnosed obstructive hypertrophic cardiomyopathy, cardiac amyloidosis, or cardiac sarcoidosis |
| Complex congenital heart disease |

**Supplementary Table S1. Exclusion criteria.**

COPD, chronic obstructive pulmonary disease; eGFR, estimated glomerular filtration rate; FEV₁, forced expiratory volume in one second; FEV₁/FVC, ratio of forced expiratory volume in one second to forced vital capacity; FVC, forced vital capacity.

| Variables | P-value | | |
| --- | --- | --- | --- |
|  | **Control vs HFpEF** | **Control vs HFrEF** | **HFpEF vs HFrEF** |
| Age (years) | 3.86E-04 | 2.42E-01 | 1.01E-04 |
| BMI (kg/m²) | 1.77E-03 | 8.45E-03 | 4.61E-01 |
| SBP (mmHg) | 3.35E-04 | 1.48E-10 | 8.00E-05 |
| DBP (mmHg) | 3.98E-04 | 5.85E-10 | 1.54E-02 |
| H_2_FPEF score | 6.91E-12 | 3.90E-08 | 3.76E-03 |
| Hypertension | 5.29E-03 | 6.22E-07 | 1.97E-02 |
| Dyslipidemia | 8.79E-04 | 5.46E-03 | 5.72E-01 |
| Atrial fibrillation | 3.16E-12 | 2.51E-06 | 1.17E-02 |
| Previous Stroke | 7.40E-03 | 1.99E-01 | 1.40E-01 |
| CCB | 1.26E-04 | 5.44E-12 | 7.14E-04 |
| BB | 5.71E-11 | 4.45E-19 | 1.29E-03 |
| MRA | 9.93E-11 | 5.85E-25 | 1.61E-06 |
| ACE-I/ARB | 1.78E-06 | 2.08E-04 | 2.58E-01 |
| ARNI | 8.90E-07 | 4.05E-09 | 2.93E-01 |
| SGLT2-I | 2.87E-16 | 2.64E-19 | 3.14E-01 |
| Diuretics | 9.64E-13 | 1.49E-17 | 8.66E-02 |
| sGC | 4.81E-01 | 3.89E-04 | 2.85E-03 |
| BNP (pg/mL) | 2.06E-21 | 1.47E-19 | 8.58E-01 |
| eGFR (mL/min/1.73m²) | 8.38E-11 | 1.50E-09 | 2.36E-01 |
| Hb (g/dL) | 1.19E-01 | 1.63E-01 | 7.32E-03 |
| LDL-C (mg/dL) | 5.95E-04 | 2.42E-03 | 7.36E-01 |
| HDL-C (mg/dL) | 1.55E-01 | 1.88E-03 | 9.57E-02 |
| TG (mg/dL) | 2.95E-01 | 3.74E-02 | 4.70E-03 |
| LVEF (%) | 1.50E-09 | 1.89E-26 | 6.83E-26 |
| E/A | 1.61E-03 | 5.32E-01 | 4.00E-02 |
| E/e' | 2.87E-04 | 7.69E-04 | 7.99E-01 |
| LVDd (mm) | 2.16E-03 | 1.85E-22 | 1.46E-15 |
| LVDs (mm) | 6.13E-05 | 4.13E-26 | 5.53E-22 |
| IVSd (mm) | 3.44E-01 | 1.23E-03 | 5.82E-05 |
| PWd (mm) | 2.78E-01 | 1.04E-02 | 1.29E-03 |
| LAD (mm) | 2.18E-09 | 1.61E-05 | 1.13E-01 |
| LAVI (mL/m^2^) | 3.69E-10 | 4.49E-10 | 4.78E-01 |

**Supplementary Table S2. Pairwise tests for baseline characteristics.**

ACE-I, angiotensin-converting enzyme inhibitor; ARB, angiotensin II receptor blocker; ARNI, angiotensin receptor–neprilysin inhibitor; BB, beta-blocker; BMI, body mass index; BNP, B-type natriuretic peptide; CCB, calcium channel blocker; DBP, diastolic blood pressure; E/A, early-to-late diastolic mitral inflow velocity ratio; E/e′, early diastolic mitral inflow velocity to mitral annular velocity ratio; eGFR, estimated glomerular filtration rate; Hb, hemoglobin; HDL-C, high-density lipoprotein cholesterol; HF, heart failure; HFpEF, heart failure with preserved ejection fraction; HFrEF, heart failure with reduced ejection fraction; IVSd, interventricular septal thickness at diastole; LAD, left atrial diameter; LAVI, left atrial volume index; LDL-C, low-density lipoprotein cholesterol; LVDd, left ventricular end-diastolic diameter; LVDs, left ventricular end-systolic diameter; LVEF, left ventricular ejection fraction; MRA, mineralocorticoid receptor antagonist; PWd, posterior wall thickness at diastole; SBP, systolic blood pressure; sGC, soluble guanylate cyclase; SGLT2-I, sodium–glucose cotransporter 2 inhibitor; TG, triglycerides.

| comparison | sex | miRNA | baseMean | log2FC | pvalue | padj |
| --- | --- | --- | --- | --- | --- | --- |
| HFpEF vs Control | female | hsa-miR-877-5p | 42.1 | -0.528 | 3.02E-04 | 6.91E-02 |
| HFpEF vs Control | female | hsa-miR-3940-3p | 171.7 | -0.685 | 6.07E-04 | 6.95E-02 |
| HFpEF vs Control | female | hsa-miR-1275 | 12.8 | -0.734 | 1.61E-03 | 9.20E-02 |
| HFpEF vs Control | female | hsa-miR-486-5p | 1955394.8 | -0.483 | 1.54E-03 | 9.20E-02 |
| HFpEF vs Control | female | hsa-miR-221-3p | 24.6 | 0.630 | 4.09E-03 | 1.87E-01 |
| HFpEF vs Control | female | hsa-miR-106b-3p | 95.9 | 0.472 | 6.79E-03 | 2.38E-01 |
| HFpEF vs Control | female | hsa-miR-30c-1-3p | 36.5 | -0.383 | 7.27E-03 | 2.38E-01 |
| HFpEF vs Control | female | hsa-miR-579-5p | 8.0 | 0.457 | 1.04E-02 | 2.98E-01 |
| HFpEF vs Control | female | hsa-miR-363-3p | 27.5 | 0.520 | 1.82E-02 | 3.30E-01 |
| HFpEF vs Control | female | hsa-miR-6803-3p | 65.1 | -0.439 | 1.35E-02 | 3.30E-01 |
| HFpEF vs Control | female | hsa-miR-139-5p | 169.3 | -0.392 | 1.87E-02 | 3.30E-01 |
| HFpEF vs Control | female | hsa-miR-425-5p | 2044.4 | 0.358 | 1.47E-02 | 3.30E-01 |
| HFpEF vs Control | female | hsa-miR-3605-3p | 879.7 | -0.342 | 1.70E-02 | 3.30E-01 |
| HFpEF vs Control | female | hsa-miR-142-3p | 42.2 | -0.669 | 2.32E-02 | 3.32E-01 |
| HFpEF vs Control | female | hsa-miR-5010-3p | 10.1 | -0.521 | 2.06E-02 | 3.32E-01 |
| HFpEF vs Control | female | hsa-miR-320c | 27.9 | -0.497 | 2.24E-02 | 3.32E-01 |
| HFpEF vs Control | female | hsa-miR-100-5p | 56.5 | 1.258 | 2.99E-02 | 3.80E-01 |
| HFpEF vs Control | female | hsa-miR-22-5p | 13.6 | 0.413 | 2.98E-02 | 3.80E-01 |
| HFpEF vs Control | female | hsa-miR-339-3p | 215.8 | 0.264 | 3.39E-02 | 4.08E-01 |
| HFpEF vs Control | female | hsa-miR-145-5p | 15.2 | -0.384 | 3.65E-02 | 4.18E-01 |
| HFpEF vs Control | female | hsa-miR-320b | 160.5 | -0.363 | 4.16E-02 | 4.54E-01 |
| HFpEF vs Control | female | hsa-miR-320d | 9.1 | -0.342 | 5.56E-02 | 4.54E-01 |
| HFpEF vs Control | female | hsa-miR-6511b-3p | 29.4 | -0.333 | 5.42E-02 | 4.54E-01 |
| HFpEF vs Control | female | hsa-miR-22-3p | 754.9 | 0.299 | 5.01E-02 | 4.54E-01 |
| HFpEF vs Control | female | hsa-miR-103a-3p | 4001.1 | 0.283 | 4.88E-02 | 4.54E-01 |
| HFpEF vs Control | female | hsa-miR-378a-3p | 136.0 | 0.229 | 5.16E-02 | 4.54E-01 |
| HFpEF vs Control | female | hsa-miR-185-5p | 21707.6 | 0.229 | 4.78E-02 | 4.54E-01 |
| HFpEF vs Control | female | hsa-miR-1301-3p | 67.5 | -0.198 | 4.94E-02 | 4.54E-01 |
| HFpEF vs Control | female | hsa-miR-98-5p | 50.6 | 0.382 | 5.81E-02 | 4.59E-01 |
| HFpEF vs Control | female | hsa-miR-15a-5p | 51.1 | 0.519 | 6.25E-02 | 4.65E-01 |
| HFpEF vs Control | female | hsa-miR-6750-5p | 8.1 | -0.373 | 6.39E-02 | 4.65E-01 |
| HFpEF vs Control | female | hsa-miR-3173-5p | 9.4 | -0.364 | 6.70E-02 | 4.65E-01 |
| HFpEF vs Control | female | hsa-miR-30e-3p | 380.9 | -0.357 | 6.67E-02 | 4.65E-01 |
| HFpEF vs Control | female | hsa-miR-17-3p | 88.2 | 0.298 | 7.19E-02 | 4.85E-01 |
| HFpEF vs Control | female | hsa-miR-4433b-5p | 23.0 | -0.377 | 8.87E-02 | 5.17E-01 |
| HFpEF vs Control | female | hsa-let-7d-3p | 140.7 | -0.343 | 9.02E-02 | 5.17E-01 |
| HFpEF vs Control | female | hsa-miR-320a-3p | 11901.0 | -0.296 | 8.16E-02 | 5.17E-01 |
| HFpEF vs Control | female | hsa-miR-150-5p | 1393.9 | -0.273 | 7.91E-02 | 5.17E-01 |
| HFpEF vs Control | female | hsa-miR-193a-5p | 55.8 | -0.248 | 8.72E-02 | 5.17E-01 |
| HFpEF vs Control | female | hsa-miR-186-5p | 206.1 | 0.200 | 8.45E-02 | 5.17E-01 |
| HFpEF vs Control | female | hsa-miR-335-5p | 68.0 | -0.468 | 1.07E-01 | 5.21E-01 |
| HFpEF vs Control | female | hsa-miR-3135b | 13.2 | -0.358 | 1.11E-01 | 5.21E-01 |
| HFpEF vs Control | female | hsa-miR-6820-3p | 12.4 | -0.337 | 1.08E-01 | 5.21E-01 |
| HFpEF vs Control | female | hsa-miR-500a-3p | 10.1 | 0.321 | 1.06E-01 | 5.21E-01 |
| HFpEF vs Control | female | hsa-miR-1260a | 27.3 | -0.279 | 1.11E-01 | 5.21E-01 |
| HFpEF vs Control | female | hsa-miR-30e-5p | 124.1 | -0.274 | 1.04E-01 | 5.21E-01 |
| HFpEF vs Control | female | hsa-miR-194-5p | 28.7 | 0.273 | 1.01E-01 | 5.21E-01 |
| HFpEF vs Control | female | hsa-let-7b-3p | 19.7 | -0.244 | 1.08E-01 | 5.21E-01 |
| HFpEF vs Control | female | hsa-miR-342-5p | 180.5 | -0.188 | 1.11E-01 | 5.21E-01 |
| HFpEF vs Control | female | hsa-miR-92b-3p | 579.4 | -0.261 | 1.16E-01 | 5.30E-01 |
| HFpEF vs Control | female | hsa-miR-1908-5p | 10.8 | -0.332 | 1.35E-01 | 5.49E-01 |
| HFpEF vs Control | female | hsa-miR-6783-3p | 14.7 | -0.297 | 1.26E-01 | 5.49E-01 |
| HFpEF vs Control | female | hsa-let-7c-5p | 83.7 | -0.230 | 1.30E-01 | 5.49E-01 |
| HFpEF vs Control | female | hsa-miR-362-5p | 47.1 | 0.207 | 1.37E-01 | 5.49E-01 |
| HFpEF vs Control | female | hsa-miR-191-3p | 21.5 | -0.206 | 1.36E-01 | 5.49E-01 |
| HFpEF vs Control | female | hsa-miR-140-3p | 2594.7 | 0.177 | 1.28E-01 | 5.49E-01 |
| HFpEF vs Control | female | hsa-miR-324-5p | 202.2 | 0.173 | 1.33E-01 | 5.49E-01 |
| HFpEF vs Control | female | hsa-miR-500a-5p | 7.5 | 0.302 | 1.46E-01 | 5.56E-01 |
| HFpEF vs Control | female | hsa-miR-339-5p | 320.9 | 0.251 | 1.45E-01 | 5.56E-01 |
| HFpEF vs Control | female | hsa-miR-107 | 930.1 | 0.243 | 1.45E-01 | 5.56E-01 |
| HFpEF vs Control | female | hsa-miR-574-5p | 97.7 | -0.271 | 1.52E-01 | 5.62E-01 |
| HFpEF vs Control | female | hsa-miR-30c-5p | 268.5 | 0.189 | 1.52E-01 | 5.62E-01 |
| HFpEF vs Control | female | hsa-miR-4301 | 8.4 | -0.357 | 1.61E-01 | 5.80E-01 |
| HFpEF vs Control | female | hsa-miR-15b-5p | 4287.6 | 0.303 | 1.62E-01 | 5.80E-01 |
| HFpEF vs Control | female | hsa-miR-99a-5p | 9.9 | 0.360 | 1.73E-01 | 5.91E-01 |
| HFpEF vs Control | female | hsa-miR-2110 | 809.0 | -0.187 | 1.72E-01 | 5.91E-01 |
| HFpEF vs Control | female | hsa-miR-423-5p | 17295.5 | -0.181 | 1.78E-01 | 5.91E-01 |
| HFpEF vs Control | female | hsa-miR-4732-5p | 277.2 | -0.165 | 1.77E-01 | 5.91E-01 |
| HFpEF vs Control | female | hsa-miR-181a-2-3p | 311.3 | 0.118 | 1.76E-01 | 5.91E-01 |
| HFpEF vs Control | female | hsa-miR-29a-3p | 104.2 | 0.222 | 1.83E-01 | 5.99E-01 |
| HFpEF vs Control | female | hsa-miR-30b-5p | 66.6 | 0.217 | 1.91E-01 | 6.06E-01 |
| HFpEF vs Control | female | hsa-miR-6511a-3p | 71.7 | -0.205 | 1.90E-01 | 6.06E-01 |
| HFpEF vs Control | female | hsa-miR-627-5p | 9.0 | -0.297 | 2.53E-01 | 6.10E-01 |
| HFpEF vs Control | female | hsa-miR-3074-5p | 12.6 | -0.285 | 3.18E-01 | 6.10E-01 |
| HFpEF vs Control | female | hsa-miR-142-5p | 3277.0 | -0.277 | 2.25E-01 | 6.10E-01 |
| HFpEF vs Control | female | hsa-miR-125b-5p | 161.0 | 0.275 | 2.88E-01 | 6.10E-01 |
| HFpEF vs Control | female | hsa-miR-4508 | 103.8 | -0.258 | 2.88E-01 | 6.10E-01 |
| HFpEF vs Control | female | hsa-miR-181a-3p | 9.9 | 0.257 | 2.26E-01 | 6.10E-01 |
| HFpEF vs Control | female | hsa-miR-6857-3p | 7.3 | -0.248 | 2.06E-01 | 6.10E-01 |
| HFpEF vs Control | female | hsa-miR-11401 | 8.4 | 0.247 | 2.43E-01 | 6.10E-01 |
| HFpEF vs Control | female | hsa-miR-10527-5p | 6.5 | 0.239 | 2.90E-01 | 6.10E-01 |
| HFpEF vs Control | female | hsa-miR-3667-5p | 15.9 | 0.237 | 3.23E-01 | 6.10E-01 |
| HFpEF vs Control | female | hsa-miR-5010-5p | 19.9 | -0.226 | 2.79E-01 | 6.10E-01 |
| HFpEF vs Control | female | hsa-miR-4685-3p | 31.3 | -0.223 | 2.99E-01 | 6.10E-01 |
| HFpEF vs Control | female | hsa-miR-324-3p | 29.9 | 0.222 | 2.07E-01 | 6.10E-01 |
| HFpEF vs Control | female | hsa-let-7g-5p | 5398.4 | 0.215 | 2.18E-01 | 6.10E-01 |
| HFpEF vs Control | female | hsa-miR-3615 | 1813.6 | -0.214 | 2.89E-01 | 6.10E-01 |
| HFpEF vs Control | female | hsa-miR-451a | 849.0 | 0.210 | 2.12E-01 | 6.10E-01 |
| HFpEF vs Control | female | hsa-miR-4800-3p | 52.2 | -0.206 | 3.21E-01 | 6.10E-01 |
| HFpEF vs Control | female | hsa-miR-1180-3p | 734.8 | -0.205 | 2.34E-01 | 6.10E-01 |
| HFpEF vs Control | female | hsa-miR-1470 | 15.0 | 0.205 | 3.07E-01 | 6.10E-01 |
| HFpEF vs Control | female | hsa-miR-1976 | 11.9 | -0.198 | 3.25E-01 | 6.10E-01 |
| HFpEF vs Control | female | hsa-miR-491-5p | 12.0 | -0.198 | 2.08E-01 | 6.10E-01 |
| HFpEF vs Control | female | hsa-let-7f-5p | 3188.5 | 0.198 | 3.09E-01 | 6.10E-01 |
| HFpEF vs Control | female | hsa-miR-345-5p | 41.4 | 0.195 | 2.35E-01 | 6.10E-01 |
| HFpEF vs Control | female | hsa-miR-941 | 215.1 | 0.193 | 2.82E-01 | 6.10E-01 |
| HFpEF vs Control | female | hsa-miR-6802-3p | 8.2 | -0.193 | 3.12E-01 | 6.10E-01 |
| HFpEF vs Control | female | hsa-let-7b-5p | 48253.9 | -0.191 | 2.92E-01 | 6.10E-01 |
| HFpEF vs Control | female | hsa-miR-1260b | 179.9 | -0.184 | 2.95E-01 | 6.10E-01 |
| HFpEF vs Control | female | hsa-miR-6877-5p | 9.3 | -0.180 | 3.24E-01 | 6.10E-01 |
| HFpEF vs Control | female | hsa-miR-6734-5p | 16.5 | -0.180 | 3.19E-01 | 6.10E-01 |
| HFpEF vs Control | female | hsa-miR-151b | 33.7 | -0.180 | 2.86E-01 | 6.10E-01 |
| HFpEF vs Control | female | hsa-miR-331-3p | 22.8 | -0.179 | 2.54E-01 | 6.10E-01 |
| HFpEF vs Control | female | hsa-miR-25-5p | 53.2 | -0.174 | 2.41E-01 | 6.10E-01 |
| HFpEF vs Control | female | hsa-miR-3200-3p | 9.3 | 0.174 | 3.24E-01 | 6.10E-01 |
| HFpEF vs Control | female | hsa-miR-636 | 66.9 | -0.173 | 2.69E-01 | 6.10E-01 |
| HFpEF vs Control | female | hsa-miR-28-3p | 172.2 | -0.171 | 2.71E-01 | 6.10E-01 |
| HFpEF vs Control | female | hsa-miR-937-3p | 29.2 | -0.162 | 2.41E-01 | 6.10E-01 |
| HFpEF vs Control | female | hsa-miR-361-5p | 183.5 | -0.159 | 2.34E-01 | 6.10E-01 |
| HFpEF vs Control | female | hsa-miR-23a-3p | 95.2 | 0.159 | 2.86E-01 | 6.10E-01 |
| HFpEF vs Control | female | hsa-miR-505-5p | 411.7 | -0.157 | 2.46E-01 | 6.10E-01 |
| HFpEF vs Control | female | hsa-miR-501-3p | 26.8 | 0.154 | 2.48E-01 | 6.10E-01 |
| HFpEF vs Control | female | hsa-miR-505-3p | 13.8 | -0.147 | 3.21E-01 | 6.10E-01 |
| HFpEF vs Control | female | hsa-miR-125a-5p | 215.7 | -0.142 | 3.09E-01 | 6.10E-01 |
| HFpEF vs Control | female | hsa-miR-1226-3p | 100.8 | -0.141 | 3.17E-01 | 6.10E-01 |
| HFpEF vs Control | female | hsa-miR-6513-3p | 58.2 | 0.137 | 2.89E-01 | 6.10E-01 |
| HFpEF vs Control | female | hsa-miR-744-5p | 436.1 | -0.134 | 2.39E-01 | 6.10E-01 |
| HFpEF vs Control | female | hsa-miR-1292-5p | 49.5 | -0.123 | 2.78E-01 | 6.10E-01 |
| HFpEF vs Control | female | hsa-miR-128-3p | 832.3 | 0.120 | 3.05E-01 | 6.10E-01 |
| HFpEF vs Control | female | hsa-miR-532-5p | 360.2 | 0.120 | 3.02E-01 | 6.10E-01 |
| HFpEF vs Control | female | hsa-miR-425-3p | 266.5 | 0.116 | 2.62E-01 | 6.10E-01 |
| HFpEF vs Control | female | hsa-miR-25-3p | 5146.4 | 0.113 | 2.63E-01 | 6.10E-01 |
| HFpEF vs Control | female | hsa-miR-409-3p | 71.1 | -0.242 | 3.35E-01 | 6.11E-01 |
| HFpEF vs Control | female | hsa-miR-200c-3p | 8.0 | 0.205 | 3.37E-01 | 6.11E-01 |
| HFpEF vs Control | female | hsa-miR-576-5p | 126.9 | 0.191 | 3.39E-01 | 6.11E-01 |
| HFpEF vs Control | female | hsa-miR-4454 | 31.3 | 0.188 | 3.36E-01 | 6.11E-01 |
| HFpEF vs Control | female | hsa-miR-671-3p | 25.2 | -0.165 | 3.30E-01 | 6.11E-01 |
| HFpEF vs Control | female | hsa-miR-197-3p | 176.7 | -0.150 | 3.44E-01 | 6.15E-01 |
| HFpEF vs Control | female | hsa-miR-660-5p | 36.8 | 0.174 | 3.50E-01 | 6.18E-01 |
| HFpEF vs Control | female | hsa-miR-93-3p | 1034.9 | 0.130 | 3.51E-01 | 6.18E-01 |
| HFpEF vs Control | female | hsa-miR-4659a-3p | 11.3 | -0.181 | 3.56E-01 | 6.21E-01 |
| HFpEF vs Control | female | hsa-miR-342-3p | 1707.3 | -0.119 | 3.58E-01 | 6.21E-01 |
| HFpEF vs Control | female | hsa-miR-18a-3p | 12.4 | 0.194 | 3.68E-01 | 6.23E-01 |
| HFpEF vs Control | female | hsa-miR-1843 | 21.2 | -0.190 | 3.70E-01 | 6.23E-01 |
| HFpEF vs Control | female | hsa-miR-1285-3p | 13.6 | 0.188 | 3.65E-01 | 6.23E-01 |
| HFpEF vs Control | female | hsa-miR-326 | 36.9 | -0.147 | 3.70E-01 | 6.23E-01 |
| HFpEF vs Control | female | hsa-miR-191-5p | 254891.5 | -0.110 | 3.73E-01 | 6.23E-01 |
| HFpEF vs Control | female | hsa-miR-424-3p | 153.8 | -0.120 | 3.78E-01 | 6.28E-01 |
| HFpEF vs Control | female | hsa-miR-26b-5p | 496.4 | -0.186 | 3.88E-01 | 6.39E-01 |
| HFpEF vs Control | female | hsa-miR-99b-5p | 142.6 | -0.147 | 3.94E-01 | 6.40E-01 |
| HFpEF vs Control | female | hsa-miR-3688-3p | 14.8 | 0.140 | 3.92E-01 | 6.40E-01 |
| HFpEF vs Control | female | hsa-miR-192-5p | 47.6 | 0.143 | 4.04E-01 | 6.52E-01 |
| HFpEF vs Control | female | hsa-miR-5189-3p | 27.8 | -0.502 | 4.14E-01 | 6.63E-01 |
| HFpEF vs Control | female | hsa-miR-629-5p | 396.1 | -0.097 | 4.17E-01 | 6.64E-01 |
| HFpEF vs Control | female | hsa-let-7d-5p | 6233.1 | 0.104 | 4.35E-01 | 6.87E-01 |
| HFpEF vs Control | female | hsa-miR-1287-5p | 13.5 | 0.171 | 4.51E-01 | 7.05E-01 |
| HFpEF vs Control | female | hsa-miR-1229-3p | 9.9 | -0.128 | 4.53E-01 | 7.05E-01 |
| HFpEF vs Control | female | hsa-miR-532-3p | 1253.4 | -0.093 | 4.65E-01 | 7.20E-01 |
| HFpEF vs Control | female | hsa-miR-10a-5p | 27.1 | -0.165 | 4.73E-01 | 7.24E-01 |
| HFpEF vs Control | female | hsa-miR-4466 | 9.0 | -0.161 | 4.74E-01 | 7.24E-01 |
| HFpEF vs Control | female | hsa-miR-4732-3p | 1022.5 | -0.111 | 4.88E-01 | 7.40E-01 |
| HFpEF vs Control | female | hsa-miR-143-3p | 10.4 | 0.146 | 4.96E-01 | 7.47E-01 |
| HFpEF vs Control | female | hsa-miR-130b-3p | 323.2 | 0.082 | 5.02E-01 | 7.52E-01 |
| HFpEF vs Control | female | hsa-miR-17-5p | 12.0 | 0.161 | 5.21E-01 | 7.65E-01 |
| HFpEF vs Control | female | hsa-miR-155-5p | 49.7 | -0.087 | 5.21E-01 | 7.65E-01 |
| HFpEF vs Control | female | hsa-miR-24-3p | 281.9 | 0.074 | 5.19E-01 | 7.65E-01 |
| HFpEF vs Control | female | hsa-miR-29b-3p | 57.5 | 0.152 | 5.34E-01 | 7.77E-01 |
| HFpEF vs Control | female | hsa-miR-20b-5p | 197.5 | 0.132 | 5.39E-01 | 7.77E-01 |
| HFpEF vs Control | female | hsa-miR-15b-3p | 176.1 | 0.131 | 5.46E-01 | 7.77E-01 |
| HFpEF vs Control | female | hsa-miR-3124-5p | 9.7 | -0.119 | 5.44E-01 | 7.77E-01 |
| HFpEF vs Control | female | hsa-miR-3157-5p | 10.8 | -0.116 | 5.41E-01 | 7.77E-01 |
| HFpEF vs Control | female | hsa-miR-7-5p | 109.9 | 0.135 | 5.74E-01 | 7.89E-01 |
| HFpEF vs Control | female | hsa-miR-16-5p | 6068.8 | 0.118 | 5.72E-01 | 7.89E-01 |
| HFpEF vs Control | female | hsa-miR-628-3p | 12.2 | -0.116 | 5.73E-01 | 7.89E-01 |
| HFpEF vs Control | female | hsa-miR-4723-5p | 8.0 | -0.111 | 5.75E-01 | 7.89E-01 |
| HFpEF vs Control | female | hsa-miR-130b-5p | 13.4 | -0.107 | 5.66E-01 | 7.89E-01 |
| HFpEF vs Control | female | hsa-let-7a-5p | 29300.0 | 0.078 | 5.71E-01 | 7.89E-01 |
| HFpEF vs Control | female | hsa-miR-126-5p | 32.6 | -0.217 | 5.93E-01 | 7.95E-01 |
| HFpEF vs Control | female | hsa-miR-20a-5p | 52.2 | -0.140 | 6.01E-01 | 7.95E-01 |
| HFpEF vs Control | female | hsa-miR-126-3p | 402.2 | 0.139 | 6.20E-01 | 7.95E-01 |
| HFpEF vs Control | female | hsa-miR-1255b-5p | 22.0 | -0.100 | 6.20E-01 | 7.95E-01 |
| HFpEF vs Control | female | hsa-let-7i-5p | 10985.9 | 0.093 | 5.86E-01 | 7.95E-01 |
| HFpEF vs Control | female | hsa-miR-10399-3p | 10.9 | -0.087 | 6.09E-01 | 7.95E-01 |
| HFpEF vs Control | female | hsa-miR-629-3p | 21.1 | -0.087 | 6.01E-01 | 7.95E-01 |
| HFpEF vs Control | female | hsa-miR-23b-3p | 27.6 | 0.081 | 6.00E-01 | 7.95E-01 |
| HFpEF vs Control | female | hsa-miR-92a-3p | 16607.0 | -0.075 | 6.12E-01 | 7.95E-01 |
| HFpEF vs Control | female | hsa-miR-181b-5p | 33.3 | 0.074 | 6.21E-01 | 7.95E-01 |
| HFpEF vs Control | female | hsa-miR-7977 | 71.8 | 0.074 | 5.90E-01 | 7.95E-01 |
| HFpEF vs Control | female | hsa-miR-151a-3p | 716.4 | 0.073 | 6.10E-01 | 7.95E-01 |
| HFpEF vs Control | female | hsa-miR-589-5p | 131.3 | -0.053 | 6.29E-01 | 8.00E-01 |
| HFpEF vs Control | female | hsa-miR-1294 | 122.5 | 0.056 | 6.33E-01 | 8.01E-01 |
| HFpEF vs Control | female | hsa-miR-182-5p | 1155.7 | -0.090 | 6.53E-01 | 8.08E-01 |
| HFpEF vs Control | female | hsa-miR-501-5p | 10.2 | 0.077 | 6.47E-01 | 8.08E-01 |
| HFpEF vs Control | female | hsa-miR-423-3p | 205.7 | 0.072 | 6.52E-01 | 8.08E-01 |
| HFpEF vs Control | female | hsa-miR-223-3p | 2633.3 | 0.055 | 6.52E-01 | 8.08E-01 |
| HFpEF vs Control | female | hsa-miR-181a-5p | 327.9 | -0.077 | 6.69E-01 | 8.19E-01 |
| HFpEF vs Control | female | hsa-miR-574-3p | 155.7 | 0.055 | 6.68E-01 | 8.19E-01 |
| HFpEF vs Control | female | hsa-miR-3200-5p | 17.8 | 0.088 | 6.78E-01 | 8.26E-01 |
| HFpEF vs Control | female | hsa-miR-106b-5p | 12.9 | 0.092 | 6.87E-01 | 8.31E-01 |
| HFpEF vs Control | female | hsa-miR-151a-5p | 9475.5 | -0.054 | 6.89E-01 | 8.31E-01 |
| HFpEF vs Control | female | hsa-miR-18b-3p | 8.8 | 0.090 | 7.02E-01 | 8.40E-01 |
| HFpEF vs Control | female | hsa-miR-183-3p | 96.3 | -0.057 | 7.06E-01 | 8.40E-01 |
| HFpEF vs Control | female | hsa-miR-361-3p | 38.4 | 0.043 | 7.08E-01 | 8.40E-01 |
| HFpEF vs Control | female | hsa-miR-3179 | 15.7 | -0.069 | 7.31E-01 | 8.50E-01 |
| HFpEF vs Control | female | hsa-miR-210-3p | 169.8 | 0.051 | 7.24E-01 | 8.50E-01 |
| HFpEF vs Control | female | hsa-miR-550a-5p | 74.8 | -0.046 | 7.28E-01 | 8.50E-01 |
| HFpEF vs Control | female | hsa-miR-30d-5p | 2251.4 | -0.045 | 7.28E-01 | 8.50E-01 |
| HFpEF vs Control | female | hsa-miR-130a-3p | 619.2 | 0.060 | 7.43E-01 | 8.55E-01 |
| HFpEF vs Control | female | hsa-miR-328-3p | 43.8 | -0.056 | 7.42E-01 | 8.55E-01 |
| HFpEF vs Control | female | hsa-miR-92b-5p | 15.5 | -0.052 | 7.76E-01 | 8.88E-01 |
| HFpEF vs Control | female | hsa-miR-122-5p | 14.1 | 0.106 | 7.82E-01 | 8.90E-01 |
| HFpEF vs Control | female | hsa-miR-7706 | 48.6 | 0.038 | 7.93E-01 | 8.99E-01 |
| HFpEF vs Control | female | hsa-miR-1270 | 26.3 | -0.052 | 8.06E-01 | 9.09E-01 |
| HFpEF vs Control | female | hsa-miR-323b-3p | 10.2 | 0.058 | 8.60E-01 | 9.60E-01 |
| HFpEF vs Control | female | hsa-miR-4448 | 13.5 | -0.029 | 8.64E-01 | 9.60E-01 |
| HFpEF vs Control | female | hsa-miR-421 | 46.7 | 0.027 | 8.72E-01 | 9.60E-01 |
| HFpEF vs Control | female | hsa-miR-652-3p | 578.3 | 0.024 | 8.69E-01 | 9.60E-01 |
| HFpEF vs Control | female | hsa-miR-3158-5p | 32.0 | 0.023 | 8.58E-01 | 9.60E-01 |
| HFpEF vs Control | female | hsa-miR-550a-3-5p | 35.8 | -0.021 | 8.76E-01 | 9.60E-01 |
| HFpEF vs Control | female | hsa-miR-29c-3p | 11.3 | 0.034 | 8.88E-01 | 9.66E-01 |
| HFpEF vs Control | female | hsa-miR-484 | 1563.8 | 0.027 | 8.90E-01 | 9.66E-01 |
| HFpEF vs Control | female | hsa-let-7i-3p | 11.1 | -0.024 | 8.96E-01 | 9.67E-01 |
| HFpEF vs Control | female | hsa-miR-93-5p | 8445.0 | 0.023 | 8.99E-01 | 9.67E-01 |
| HFpEF vs Control | female | hsa-miR-21-5p | 12.6 | -0.025 | 9.22E-01 | 9.68E-01 |
| HFpEF vs Control | female | hsa-miR-503-5p | 24.8 | -0.020 | 9.20E-01 | 9.68E-01 |
| HFpEF vs Control | female | hsa-miR-3928-3p | 9.8 | 0.020 | 9.13E-01 | 9.68E-01 |
| HFpEF vs Control | female | hsa-miR-10401-3p | 7.8 | -0.020 | 9.27E-01 | 9.68E-01 |
| HFpEF vs Control | female | hsa-miR-26a-5p | 950.0 | -0.019 | 9.14E-01 | 9.68E-01 |
| HFpEF vs Control | female | hsa-miR-664a-3p | 7.7 | -0.018 | 9.37E-01 | 9.68E-01 |
| HFpEF vs Control | female | hsa-let-7e-5p | 13.3 | -0.017 | 9.38E-01 | 9.68E-01 |
| HFpEF vs Control | female | hsa-miR-942-5p | 478.2 | -0.014 | 9.31E-01 | 9.68E-01 |
| HFpEF vs Control | female | hsa-miR-183-5p | 60.7 | 0.014 | 9.38E-01 | 9.68E-01 |
| HFpEF vs Control | female | hsa-miR-664a-5p | 11.6 | 0.012 | 9.52E-01 | 9.69E-01 |
| HFpEF vs Control | female | hsa-miR-378a-5p | 18.7 | 0.011 | 9.46E-01 | 9.69E-01 |
| HFpEF vs Control | female | hsa-miR-1306-5p | 216.3 | 0.011 | 9.50E-01 | 9.69E-01 |
| HFpEF vs Control | female | hsa-miR-1304-3p | 68.2 | -0.005 | 9.63E-01 | 9.76E-01 |
| HFpEF vs Control | female | hsa-miR-3187-3p | 6.4 | -0.007 | 9.74E-01 | 9.78E-01 |
| HFpEF vs Control | female | hsa-miR-1307-3p | 72.3 | 0.006 | 9.72E-01 | 9.78E-01 |
| HFpEF vs Control | female | hsa-miR-625-5p | 387.0 | 0.002 | 9.88E-01 | 9.88E-01 |
| HFpEF vs Control | male | hsa-miR-30b-5p | 66.1 | 0.955 | 3.61E-08 | 7.59E-06 |
| HFpEF vs Control | male | hsa-miR-30c-5p | 282.0 | 0.720 | 6.62E-08 | 7.59E-06 |
| HFpEF vs Control | male | hsa-miR-29b-3p | 64.3 | 1.103 | 5.83E-07 | 4.45E-05 |
| HFpEF vs Control | male | hsa-miR-192-5p | 50.6 | 0.755 | 1.50E-06 | 8.58E-05 |
| HFpEF vs Control | male | hsa-miR-23a-3p | 100.9 | 0.668 | 3.28E-06 | 1.50E-04 |
| HFpEF vs Control | male | hsa-miR-128-3p | 868.6 | 0.562 | 4.34E-06 | 1.66E-04 |
| HFpEF vs Control | male | hsa-miR-15b-3p | 186.6 | 0.913 | 8.58E-06 | 2.81E-04 |
| HFpEF vs Control | male | hsa-miR-186-5p | 239.3 | 0.524 | 1.10E-05 | 3.15E-04 |
| HFpEF vs Control | male | hsa-miR-223-3p | 2670.6 | 0.613 | 4.21E-05 | 9.41E-04 |
| HFpEF vs Control | male | hsa-miR-6803-3p | 83.2 | -0.610 | 4.52E-05 | 9.41E-04 |
| HFpEF vs Control | male | hsa-miR-22-3p | 818.2 | 0.523 | 4.00E-05 | 9.41E-04 |
| HFpEF vs Control | male | hsa-miR-4454 | 39.1 | 0.680 | 8.94E-05 | 1.71E-03 |
| HFpEF vs Control | male | hsa-miR-142-5p | 2901.9 | 0.697 | 1.09E-04 | 1.92E-03 |
| HFpEF vs Control | male | hsa-miR-10a-5p | 28.6 | 0.969 | 1.84E-04 | 2.81E-03 |
| HFpEF vs Control | male | hsa-miR-221-3p | 30.9 | 0.793 | 1.76E-04 | 2.81E-03 |
| HFpEF vs Control | male | hsa-miR-193a-5p | 70.6 | -0.501 | 2.05E-04 | 2.93E-03 |
| HFpEF vs Control | male | hsa-miR-181a-3p | 9.8 | 0.688 | 2.82E-04 | 3.80E-03 |
| HFpEF vs Control | male | hsa-miR-3940-3p | 188.6 | -0.630 | 3.70E-04 | 4.70E-03 |
| HFpEF vs Control | male | hsa-miR-451a | 886.0 | 0.591 | 3.95E-04 | 4.76E-03 |
| HFpEF vs Control | male | hsa-miR-1301-3p | 82.1 | -0.354 | 4.56E-04 | 5.22E-03 |
| HFpEF vs Control | male | hsa-miR-194-5p | 30.0 | 0.605 | 4.84E-04 | 5.28E-03 |
| HFpEF vs Control | male | hsa-let-7c-5p | 113.1 | -0.488 | 5.15E-04 | 5.36E-03 |
| HFpEF vs Control | male | hsa-miR-576-5p | 146.0 | 0.689 | 6.68E-04 | 6.33E-03 |
| HFpEF vs Control | male | hsa-miR-151a-3p | 782.2 | 0.520 | 7.09E-04 | 6.33E-03 |
| HFpEF vs Control | male | hsa-miR-425-5p | 2205.9 | 0.444 | 7.18E-04 | 6.33E-03 |
| HFpEF vs Control | male | hsa-miR-877-5p | 50.6 | -0.432 | 6.64E-04 | 6.33E-03 |
| HFpEF vs Control | male | hsa-miR-942-5p | 551.2 | 0.407 | 9.32E-04 | 7.90E-03 |
| HFpEF vs Control | male | hsa-miR-185-5p | 25801.5 | 0.334 | 1.40E-03 | 1.14E-02 |
| HFpEF vs Control | male | hsa-miR-1306-5p | 235.2 | 0.454 | 1.52E-03 | 1.20E-02 |
| HFpEF vs Control | male | hsa-miR-1180-3p | 920.9 | -0.408 | 1.73E-03 | 1.32E-02 |
| HFpEF vs Control | male | hsa-miR-181a-2-3p | 315.0 | 0.285 | 1.81E-03 | 1.34E-02 |
| HFpEF vs Control | male | hsa-miR-486-5p | 2231664.7 | -0.402 | 2.02E-03 | 1.44E-02 |
| HFpEF vs Control | male | hsa-miR-23b-3p | 29.2 | 0.400 | 2.43E-03 | 1.68E-02 |
| HFpEF vs Control | male | hsa-miR-574-3p | 178.0 | 0.396 | 2.54E-03 | 1.71E-02 |
| HFpEF vs Control | male | hsa-miR-320c | 36.1 | -0.493 | 2.96E-03 | 1.94E-02 |
| HFpEF vs Control | male | hsa-miR-4508 | 130.4 | -0.604 | 3.49E-03 | 2.05E-02 |
| HFpEF vs Control | male | hsa-let-7e-5p | 16.2 | -0.491 | 3.45E-03 | 2.05E-02 |
| HFpEF vs Control | male | hsa-miR-106b-3p | 106.3 | 0.445 | 3.43E-03 | 2.05E-02 |
| HFpEF vs Control | male | hsa-miR-1843 | 25.0 | -0.418 | 3.43E-03 | 2.05E-02 |
| HFpEF vs Control | male | hsa-miR-744-5p | 503.0 | -0.230 | 3.84E-03 | 2.20E-02 |
| HFpEF vs Control | male | hsa-miR-181a-5p | 393.7 | -0.382 | 4.03E-03 | 2.25E-02 |
| HFpEF vs Control | male | hsa-miR-4732-5p | 318.8 | -0.287 | 5.09E-03 | 2.77E-02 |
| HFpEF vs Control | male | hsa-miR-320b | 194.6 | -0.382 | 5.24E-03 | 2.79E-02 |
| HFpEF vs Control | male | hsa-miR-335-5p | 50.7 | 0.631 | 5.94E-03 | 3.09E-02 |
| HFpEF vs Control | male | hsa-miR-28-3p | 172.0 | 0.441 | 6.94E-03 | 3.53E-02 |
| HFpEF vs Control | male | hsa-miR-500a-3p | 12.8 | 0.422 | 7.20E-03 | 3.59E-02 |
| HFpEF vs Control | male | hsa-miR-7-5p | 116.1 | 0.497 | 8.66E-03 | 4.18E-02 |
| HFpEF vs Control | male | hsa-miR-320a-3p | 14787.6 | -0.349 | 8.75E-03 | 4.18E-02 |
| HFpEF vs Control | male | hsa-miR-550a-3-5p | 37.2 | -0.342 | 9.57E-03 | 4.47E-02 |
| HFpEF vs Control | male | hsa-let-7b-5p | 67530.7 | -0.403 | 1.09E-02 | 4.99E-02 |
| HFpEF vs Control | male | hsa-miR-22-5p | 15.5 | 0.401 | 1.12E-02 | 5.04E-02 |
| HFpEF vs Control | male | hsa-miR-1260b | 215.3 | -0.417 | 1.20E-02 | 5.28E-02 |
| HFpEF vs Control | male | hsa-miR-125a-5p | 224.0 | 0.342 | 1.30E-02 | 5.63E-02 |
| HFpEF vs Control | male | hsa-miR-361-3p | 39.3 | 0.256 | 1.39E-02 | 5.91E-02 |
| HFpEF vs Control | male | hsa-miR-324-5p | 212.7 | 0.287 | 1.48E-02 | 6.15E-02 |
| HFpEF vs Control | male | hsa-miR-182-5p | 1205.7 | 0.476 | 1.50E-02 | 6.15E-02 |
| HFpEF vs Control | male | hsa-miR-99a-5p | 9.7 | 0.519 | 1.55E-02 | 6.23E-02 |
| HFpEF vs Control | male | hsa-miR-342-3p | 1838.3 | 0.302 | 1.69E-02 | 6.67E-02 |
| HFpEF vs Control | male | hsa-miR-18a-3p | 13.2 | 0.440 | 1.72E-02 | 6.69E-02 |
| HFpEF vs Control | male | hsa-miR-505-3p | 15.3 | 0.377 | 1.86E-02 | 7.00E-02 |
| HFpEF vs Control | male | hsa-miR-93-3p | 1129.1 | 0.276 | 1.86E-02 | 7.00E-02 |
| HFpEF vs Control | male | hsa-miR-1260a | 31.7 | -0.399 | 1.94E-02 | 7.17E-02 |
| HFpEF vs Control | male | hsa-miR-421 | 49.5 | 0.376 | 2.14E-02 | 7.23E-02 |
| HFpEF vs Control | male | hsa-miR-3124-5p | 14.2 | -0.350 | 2.15E-02 | 7.23E-02 |
| HFpEF vs Control | male | hsa-miR-4732-3p | 1105.3 | 0.289 | 2.08E-02 | 7.23E-02 |
| HFpEF vs Control | male | hsa-miR-2110 | 1017.9 | -0.268 | 2.11E-02 | 7.23E-02 |
| HFpEF vs Control | male | hsa-miR-652-3p | 671.8 | -0.244 | 2.13E-02 | 7.23E-02 |
| HFpEF vs Control | male | hsa-miR-30d-5p | 2451.0 | -0.229 | 2.13E-02 | 7.23E-02 |
| HFpEF vs Control | male | hsa-miR-1470 | 12.0 | 0.409 | 2.22E-02 | 7.37E-02 |
| HFpEF vs Control | male | hsa-miR-339-5p | 345.6 | 0.333 | 2.25E-02 | 7.37E-02 |
| HFpEF vs Control | male | hsa-miR-98-5p | 59.3 | 0.443 | 2.31E-02 | 7.44E-02 |
| HFpEF vs Control | male | hsa-miR-151b | 35.9 | -0.324 | 2.39E-02 | 7.61E-02 |
| HFpEF vs Control | male | hsa-miR-345-5p | 43.7 | 0.261 | 2.69E-02 | 8.43E-02 |
| HFpEF vs Control | male | hsa-miR-342-5p | 209.2 | -0.213 | 2.81E-02 | 8.69E-02 |
| HFpEF vs Control | male | hsa-miR-500a-5p | 9.4 | 0.345 | 2.86E-02 | 8.73E-02 |
| HFpEF vs Control | male | hsa-miR-574-5p | 141.7 | -0.381 | 3.31E-02 | 9.96E-02 |
| HFpEF vs Control | male | hsa-miR-25-3p | 5626.8 | 0.196 | 3.46E-02 | 1.03E-01 |
| HFpEF vs Control | male | hsa-miR-142-3p | 31.4 | 0.474 | 3.55E-02 | 1.04E-01 |
| HFpEF vs Control | male | hsa-miR-664a-3p | 9.6 | 0.359 | 3.92E-02 | 1.14E-01 |
| HFpEF vs Control | male | hsa-miR-143-3p | 10.8 | 0.403 | 4.05E-02 | 1.16E-01 |
| HFpEF vs Control | male | hsa-miR-99b-5p | 147.5 | 0.316 | 4.28E-02 | 1.21E-01 |
| HFpEF vs Control | male | hsa-miR-1292-5p | 57.3 | -0.249 | 4.47E-02 | 1.25E-01 |
| HFpEF vs Control | male | hsa-miR-15a-5p | 60.7 | 0.518 | 4.54E-02 | 1.25E-01 |
| HFpEF vs Control | male | hsa-miR-4448 | 13.9 | -0.274 | 4.73E-02 | 1.29E-01 |
| HFpEF vs Control | male | hsa-miR-324-3p | 30.6 | 0.263 | 4.94E-02 | 1.33E-01 |
| HFpEF vs Control | male | hsa-miR-29a-3p | 104.9 | 0.305 | 5.07E-02 | 1.35E-01 |
| HFpEF vs Control | male | hsa-miR-3688-3p | 15.8 | 0.296 | 5.15E-02 | 1.36E-01 |
| HFpEF vs Control | male | hsa-miR-5010-3p | 11.2 | -0.370 | 5.68E-02 | 1.48E-01 |
| HFpEF vs Control | male | hsa-miR-1275 | 12.9 | -0.342 | 5.89E-02 | 1.48E-01 |
| HFpEF vs Control | male | hsa-let-7i-3p | 13.2 | -0.279 | 5.86E-02 | 1.48E-01 |
| HFpEF vs Control | male | hsa-miR-6734-5p | 20.5 | -0.272 | 5.82E-02 | 1.48E-01 |
| HFpEF vs Control | male | hsa-let-7b-3p | 20.7 | 0.263 | 6.33E-02 | 1.58E-01 |
| HFpEF vs Control | male | hsa-miR-4800-3p | 58.1 | -0.383 | 6.47E-02 | 1.59E-01 |
| HFpEF vs Control | male | hsa-miR-3615 | 2202.1 | -0.349 | 6.55E-02 | 1.59E-01 |
| HFpEF vs Control | male | hsa-let-7i-5p | 14431.1 | -0.315 | 6.66E-02 | 1.59E-01 |
| HFpEF vs Control | male | hsa-miR-140-3p | 2735.8 | 0.212 | 6.60E-02 | 1.59E-01 |
| HFpEF vs Control | male | hsa-miR-181b-5p | 38.3 | -0.212 | 6.89E-02 | 1.63E-01 |
| HFpEF vs Control | male | hsa-miR-629-5p | 515.7 | -0.188 | 7.05E-02 | 1.65E-01 |
| HFpEF vs Control | male | hsa-miR-6513-3p | 62.3 | 0.221 | 7.44E-02 | 1.72E-01 |
| HFpEF vs Control | male | hsa-miR-320d | 9.6 | -0.286 | 7.61E-02 | 1.74E-01 |
| HFpEF vs Control | male | hsa-miR-30e-5p | 135.1 | -0.266 | 7.68E-02 | 1.74E-01 |
| HFpEF vs Control | male | hsa-miR-378a-5p | 22.1 | 0.261 | 8.33E-02 | 1.87E-01 |
| HFpEF vs Control | male | hsa-miR-29c-3p | 12.6 | 0.372 | 8.75E-02 | 1.92E-01 |
| HFpEF vs Control | male | hsa-miR-3173-5p | 13.2 | -0.372 | 8.79E-02 | 1.92E-01 |
| HFpEF vs Control | male | hsa-miR-25-5p | 71.5 | -0.240 | 8.80E-02 | 1.92E-01 |
| HFpEF vs Control | male | hsa-miR-155-5p | 59.7 | -0.214 | 9.48E-02 | 2.05E-01 |
| HFpEF vs Control | male | hsa-miR-363-3p | 26.7 | 0.340 | 9.84E-02 | 2.11E-01 |
| HFpEF vs Control | male | hsa-miR-3605-3p | 1000.2 | -0.194 | 1.05E-01 | 2.19E-01 |
| HFpEF vs Control | male | hsa-miR-7977 | 77.0 | 0.169 | 1.05E-01 | 2.19E-01 |
| HFpEF vs Control | male | hsa-miR-589-5p | 152.4 | -0.166 | 1.05E-01 | 2.19E-01 |
| HFpEF vs Control | male | hsa-miR-3158-5p | 33.6 | 0.222 | 1.09E-01 | 2.23E-01 |
| HFpEF vs Control | male | hsa-miR-339-3p | 233.6 | 0.184 | 1.09E-01 | 2.23E-01 |
| HFpEF vs Control | male | hsa-miR-10399-3p | 11.1 | -0.297 | 1.14E-01 | 2.31E-01 |
| HFpEF vs Control | male | hsa-miR-501-5p | 14.4 | 0.212 | 1.18E-01 | 2.37E-01 |
| HFpEF vs Control | male | hsa-miR-424-3p | 182.0 | -0.208 | 1.23E-01 | 2.45E-01 |
| HFpEF vs Control | male | hsa-miR-579-5p | 10.0 | -0.282 | 1.28E-01 | 2.52E-01 |
| HFpEF vs Control | male | hsa-miR-200c-3p | 10.2 | -0.261 | 1.31E-01 | 2.56E-01 |
| HFpEF vs Control | male | hsa-miR-93-5p | 11444.1 | -0.277 | 1.33E-01 | 2.58E-01 |
| HFpEF vs Control | male | hsa-miR-6511b-3p | 35.6 | -0.228 | 1.40E-01 | 2.70E-01 |
| HFpEF vs Control | male | hsa-miR-409-3p | 69.5 | 0.305 | 1.48E-01 | 2.83E-01 |
| HFpEF vs Control | male | hsa-miR-4685-3p | 39.7 | -0.281 | 1.52E-01 | 2.88E-01 |
| HFpEF vs Control | male | hsa-miR-3157-5p | 12.2 | -0.253 | 1.64E-01 | 3.08E-01 |
| HFpEF vs Control | male | hsa-miR-423-5p | 19867.9 | -0.146 | 1.69E-01 | 3.15E-01 |
| HFpEF vs Control | male | hsa-let-7f-5p | 3830.5 | 0.239 | 1.80E-01 | 3.30E-01 |
| HFpEF vs Control | male | hsa-miR-1285-3p | 15.6 | 0.233 | 1.79E-01 | 3.30E-01 |
| HFpEF vs Control | male | hsa-miR-323b-3p | 9.4 | 0.418 | 1.88E-01 | 3.38E-01 |
| HFpEF vs Control | male | hsa-miR-3135b | 17.1 | -0.255 | 1.89E-01 | 3.38E-01 |
| HFpEF vs Control | male | hsa-miR-16-5p | 6625.5 | 0.236 | 1.93E-01 | 3.38E-01 |
| HFpEF vs Control | male | hsa-miR-3200-3p | 10.1 | 0.206 | 1.94E-01 | 3.38E-01 |
| HFpEF vs Control | male | hsa-miR-628-3p | 14.8 | -0.199 | 1.95E-01 | 3.38E-01 |
| HFpEF vs Control | male | hsa-miR-625-5p | 458.9 | 0.185 | 1.95E-01 | 3.38E-01 |
| HFpEF vs Control | male | hsa-miR-505-5p | 490.1 | -0.157 | 1.93E-01 | 3.38E-01 |
| HFpEF vs Control | male | hsa-miR-24-3p | 322.2 | 0.139 | 2.17E-01 | 3.73E-01 |
| HFpEF vs Control | male | hsa-miR-145-5p | 15.7 | -0.190 | 2.19E-01 | 3.74E-01 |
| HFpEF vs Control | male | hsa-miR-6750-5p | 11.1 | -0.195 | 2.22E-01 | 3.77E-01 |
| HFpEF vs Control | male | hsa-miR-627-5p | 7.8 | -0.279 | 2.33E-01 | 3.92E-01 |
| HFpEF vs Control | male | hsa-miR-191-3p | 23.5 | -0.135 | 2.34E-01 | 3.92E-01 |
| HFpEF vs Control | male | hsa-miR-3928-3p | 12.1 | -0.182 | 2.38E-01 | 3.95E-01 |
| HFpEF vs Control | male | hsa-miR-636 | 78.2 | -0.162 | 2.40E-01 | 3.95E-01 |
| HFpEF vs Control | male | hsa-miR-4301 | 9.7 | -0.257 | 2.57E-01 | 4.19E-01 |
| HFpEF vs Control | male | hsa-miR-106b-5p | 15.6 | -0.214 | 2.60E-01 | 4.19E-01 |
| HFpEF vs Control | male | hsa-miR-30c-1-3p | 39.6 | -0.150 | 2.58E-01 | 4.19E-01 |
| HFpEF vs Control | male | hsa-miR-1304-3p | 77.6 | 0.097 | 2.71E-01 | 4.34E-01 |
| HFpEF vs Control | male | hsa-miR-107 | 1150.3 | -0.172 | 2.74E-01 | 4.36E-01 |
| HFpEF vs Control | male | hsa-miR-18b-3p | 11.2 | 0.212 | 2.78E-01 | 4.38E-01 |
| HFpEF vs Control | male | hsa-miR-4723-5p | 9.6 | -0.180 | 2.92E-01 | 4.58E-01 |
| HFpEF vs Control | male | hsa-let-7d-5p | 7548.7 | -0.128 | 3.00E-01 | 4.67E-01 |
| HFpEF vs Control | male | hsa-miR-15b-5p | 4880.6 | 0.235 | 3.10E-01 | 4.79E-01 |
| HFpEF vs Control | male | hsa-miR-6783-3p | 16.3 | -0.179 | 3.30E-01 | 5.07E-01 |
| HFpEF vs Control | male | hsa-miR-331-3p | 26.4 | -0.137 | 3.35E-01 | 5.11E-01 |
| HFpEF vs Control | male | hsa-miR-532-5p | 423.3 | 0.129 | 3.37E-01 | 5.12E-01 |
| HFpEF vs Control | male | hsa-miR-423-3p | 214.3 | 0.134 | 3.41E-01 | 5.14E-01 |
| HFpEF vs Control | male | hsa-miR-1294 | 161.2 | -0.111 | 3.48E-01 | 5.21E-01 |
| HFpEF vs Control | male | hsa-miR-125b-5p | 220.9 | -0.214 | 3.55E-01 | 5.27E-01 |
| HFpEF vs Control | male | hsa-miR-20a-5p | 65.2 | -0.234 | 3.75E-01 | 5.54E-01 |
| HFpEF vs Control | male | hsa-miR-3179 | 20.0 | 0.138 | 3.98E-01 | 5.84E-01 |
| HFpEF vs Control | male | hsa-miR-1229-3p | 12.4 | -0.137 | 4.18E-01 | 6.09E-01 |
| HFpEF vs Control | male | hsa-miR-5189-3p | 33.5 | -0.454 | 4.27E-01 | 6.19E-01 |
| HFpEF vs Control | male | hsa-miR-660-5p | 40.7 | 0.108 | 4.45E-01 | 6.38E-01 |
| HFpEF vs Control | male | hsa-let-7a-5p | 36486.6 | -0.103 | 4.44E-01 | 6.38E-01 |
| HFpEF vs Control | male | hsa-miR-21-5p | 15.1 | 0.180 | 4.62E-01 | 6.45E-01 |
| HFpEF vs Control | male | hsa-miR-3187-3p | 8.9 | -0.130 | 4.62E-01 | 6.45E-01 |
| HFpEF vs Control | male | hsa-miR-4659a-3p | 12.8 | 0.129 | 4.58E-01 | 6.45E-01 |
| HFpEF vs Control | male | hsa-miR-532-3p | 1373.5 | 0.079 | 4.59E-01 | 6.45E-01 |
| HFpEF vs Control | male | hsa-miR-4433b-5p | 24.6 | -0.142 | 4.81E-01 | 6.56E-01 |
| HFpEF vs Control | male | hsa-miR-3200-5p | 23.9 | 0.121 | 4.81E-01 | 6.56E-01 |
| HFpEF vs Control | male | hsa-miR-328-3p | 50.9 | -0.113 | 4.73E-01 | 6.56E-01 |
| HFpEF vs Control | male | hsa-miR-92b-3p | 634.3 | -0.101 | 4.81E-01 | 6.56E-01 |
| HFpEF vs Control | male | hsa-miR-4466 | 9.8 | -0.142 | 4.84E-01 | 6.56E-01 |
| HFpEF vs Control | male | hsa-miR-6820-3p | 13.9 | 0.127 | 4.91E-01 | 6.61E-01 |
| HFpEF vs Control | male | hsa-miR-1226-3p | 124.9 | 0.085 | 4.97E-01 | 6.65E-01 |
| HFpEF vs Control | male | hsa-miR-1287-5p | 19.6 | -0.141 | 5.04E-01 | 6.71E-01 |
| HFpEF vs Control | male | hsa-miR-3074-5p | 16.1 | 0.196 | 5.17E-01 | 6.85E-01 |
| HFpEF vs Control | male | hsa-miR-3667-5p | 17.7 | 0.138 | 5.32E-01 | 6.94E-01 |
| HFpEF vs Control | male | hsa-miR-26a-5p | 1038.0 | -0.098 | 5.28E-01 | 6.94E-01 |
| HFpEF vs Control | male | hsa-miR-629-3p | 23.3 | 0.078 | 5.33E-01 | 6.94E-01 |
| HFpEF vs Control | male | hsa-miR-30e-3p | 397.8 | -0.106 | 5.39E-01 | 6.97E-01 |
| HFpEF vs Control | male | hsa-miR-671-3p | 26.8 | -0.089 | 5.42E-01 | 6.97E-01 |
| HFpEF vs Control | male | hsa-miR-130a-3p | 738.1 | 0.096 | 5.46E-01 | 6.99E-01 |
| HFpEF vs Control | male | hsa-miR-197-3p | 199.7 | 0.087 | 5.59E-01 | 7.12E-01 |
| HFpEF vs Control | male | hsa-miR-6802-3p | 9.8 | 0.092 | 5.74E-01 | 7.26E-01 |
| HFpEF vs Control | male | hsa-miR-126-3p | 451.3 | 0.148 | 6.02E-01 | 7.30E-01 |
| HFpEF vs Control | male | hsa-miR-11401 | 10.0 | 0.103 | 5.86E-01 | 7.30E-01 |
| HFpEF vs Control | male | hsa-miR-484 | 1827.8 | -0.089 | 5.86E-01 | 7.30E-01 |
| HFpEF vs Control | male | hsa-miR-6877-5p | 10.7 | -0.088 | 5.88E-01 | 7.30E-01 |
| HFpEF vs Control | male | hsa-miR-92b-5p | 17.8 | -0.082 | 5.94E-01 | 7.30E-01 |
| HFpEF vs Control | male | hsa-miR-664a-5p | 15.5 | -0.071 | 6.00E-01 | 7.30E-01 |
| HFpEF vs Control | male | hsa-miR-937-3p | 32.6 | -0.070 | 5.93E-01 | 7.30E-01 |
| HFpEF vs Control | male | hsa-miR-191-5p | 284910.9 | 0.067 | 6.00E-01 | 7.30E-01 |
| HFpEF vs Control | male | hsa-miR-941 | 256.1 | 0.084 | 6.11E-01 | 7.37E-01 |
| HFpEF vs Control | male | hsa-miR-100-5p | 74.5 | -0.245 | 6.27E-01 | 7.48E-01 |
| HFpEF vs Control | male | hsa-let-7d-3p | 124.2 | 0.061 | 6.25E-01 | 7.48E-01 |
| HFpEF vs Control | male | hsa-miR-126-5p | 30.4 | 0.175 | 6.42E-01 | 7.58E-01 |
| HFpEF vs Control | male | hsa-miR-1976 | 14.4 | -0.086 | 6.39E-01 | 7.58E-01 |
| HFpEF vs Control | male | hsa-miR-17-5p | 13.7 | -0.104 | 6.45E-01 | 7.58E-01 |
| HFpEF vs Control | male | hsa-miR-550a-5p | 76.6 | 0.048 | 6.56E-01 | 7.66E-01 |
| HFpEF vs Control | male | hsa-miR-139-5p | 171.0 | -0.074 | 6.71E-01 | 7.79E-01 |
| HFpEF vs Control | male | hsa-miR-183-3p | 118.6 | -0.067 | 6.87E-01 | 7.95E-01 |
| HFpEF vs Control | male | hsa-miR-378a-3p | 171.2 | 0.044 | 6.94E-01 | 7.99E-01 |
| HFpEF vs Control | male | hsa-miR-20b-5p | 242.9 | -0.069 | 7.42E-01 | 8.44E-01 |
| HFpEF vs Control | male | hsa-miR-503-5p | 31.0 | -0.056 | 7.45E-01 | 8.44E-01 |
| HFpEF vs Control | male | hsa-miR-1270 | 35.6 | -0.056 | 7.44E-01 | 8.44E-01 |
| HFpEF vs Control | male | hsa-miR-6511a-3p | 88.0 | -0.048 | 7.48E-01 | 8.44E-01 |
| HFpEF vs Control | male | hsa-miR-26b-5p | 643.1 | -0.062 | 7.56E-01 | 8.45E-01 |
| HFpEF vs Control | male | hsa-miR-501-3p | 31.7 | 0.044 | 7.53E-01 | 8.45E-01 |
| HFpEF vs Control | male | hsa-miR-151a-5p | 10450.2 | -0.037 | 7.73E-01 | 8.60E-01 |
| HFpEF vs Control | male | hsa-miR-5010-5p | 23.6 | -0.036 | 8.00E-01 | 8.85E-01 |
| HFpEF vs Control | male | hsa-miR-1307-3p | 94.4 | -0.033 | 8.05E-01 | 8.86E-01 |
| HFpEF vs Control | male | hsa-miR-6857-3p | 5.3 | -0.044 | 8.27E-01 | 8.90E-01 |
| HFpEF vs Control | male | hsa-let-7g-5p | 6910.6 | 0.043 | 8.16E-01 | 8.90E-01 |
| HFpEF vs Control | male | hsa-miR-326 | 37.9 | 0.036 | 8.17E-01 | 8.90E-01 |
| HFpEF vs Control | male | hsa-miR-10527-5p | 9.3 | 0.034 | 8.26E-01 | 8.90E-01 |
| HFpEF vs Control | male | hsa-miR-17-3p | 104.2 | 0.029 | 8.31E-01 | 8.90E-01 |
| HFpEF vs Control | male | hsa-miR-7706 | 59.5 | -0.026 | 8.32E-01 | 8.90E-01 |
| HFpEF vs Control | male | hsa-miR-362-5p | 58.4 | 0.020 | 8.69E-01 | 9.26E-01 |
| HFpEF vs Control | male | hsa-miR-361-5p | 185.5 | -0.014 | 9.03E-01 | 9.57E-01 |
| HFpEF vs Control | male | hsa-miR-130b-5p | 14.4 | 0.019 | 9.12E-01 | 9.58E-01 |
| HFpEF vs Control | male | hsa-miR-1255b-5p | 26.2 | 0.017 | 9.08E-01 | 9.58E-01 |
| HFpEF vs Control | male | hsa-miR-210-3p | 218.7 | 0.016 | 9.20E-01 | 9.58E-01 |
| HFpEF vs Control | male | hsa-miR-130b-3p | 366.4 | 0.011 | 9.19E-01 | 9.58E-01 |
| HFpEF vs Control | male | hsa-miR-103a-3p | 4902.6 | -0.010 | 9.42E-01 | 9.73E-01 |
| HFpEF vs Control | male | hsa-miR-491-5p | 14.9 | -0.010 | 9.43E-01 | 9.73E-01 |
| HFpEF vs Control | male | hsa-miR-183-5p | 76.8 | -0.008 | 9.66E-01 | 9.83E-01 |
| HFpEF vs Control | male | hsa-miR-150-5p | 1427.3 | 0.008 | 9.62E-01 | 9.83E-01 |
| HFpEF vs Control | male | hsa-miR-425-3p | 295.6 | 0.005 | 9.66E-01 | 9.83E-01 |
| HFpEF vs Control | male | hsa-miR-10401-3p | 9.0 | -0.007 | 9.78E-01 | 9.88E-01 |
| HFpEF vs Control | male | hsa-miR-1908-5p | 13.1 | 0.005 | 9.79E-01 | 9.88E-01 |
| HFpEF vs Control | male | hsa-miR-92a-3p | 18241.1 | 0.002 | 9.89E-01 | 9.93E-01 |
| HFpEF vs Control | male | hsa-miR-122-5p | 25.2 | 0.000 | 1.00E+00 | 1.00E+00 |
| HFrEF vs Control | female | hsa-miR-1275 | 13.8 | -0.850 | 8.82E-04 | 1.26E-01 |
| HFrEF vs Control | female | hsa-miR-193a-5p | 57.3 | -0.476 | 1.10E-03 | 1.26E-01 |
| HFrEF vs Control | female | hsa-miR-335-5p | 107.1 | 1.266 | 2.57E-03 | 1.96E-01 |
| HFrEF vs Control | female | hsa-miR-142-5p | 4382.1 | 0.879 | 4.98E-03 | 2.85E-01 |
| HFrEF vs Control | female | hsa-miR-125b-5p | 194.4 | 0.652 | 3.62E-02 | 4.62E-01 |
| HFrEF vs Control | female | hsa-miR-3615 | 1853.7 | -0.552 | 1.22E-02 | 4.62E-01 |
| HFrEF vs Control | female | hsa-miR-10399-3p | 10.3 | -0.496 | 1.45E-02 | 4.62E-01 |
| HFrEF vs Control | female | hsa-miR-22-5p | 14.3 | 0.472 | 3.73E-02 | 4.62E-01 |
| HFrEF vs Control | female | hsa-miR-6783-3p | 15.5 | -0.471 | 1.94E-02 | 4.62E-01 |
| HFrEF vs Control | female | hsa-miR-320c | 30.1 | -0.466 | 3.84E-02 | 4.62E-01 |
| HFrEF vs Control | female | hsa-miR-1260a | 27.9 | -0.451 | 2.67E-02 | 4.62E-01 |
| HFrEF vs Control | female | hsa-miR-223-3p | 2867.6 | 0.407 | 2.35E-02 | 4.62E-01 |
| HFrEF vs Control | female | hsa-miR-210-3p | 194.2 | 0.384 | 3.32E-02 | 4.62E-01 |
| HFrEF vs Control | female | hsa-miR-139-5p | 177.7 | -0.380 | 3.80E-02 | 4.62E-01 |
| HFrEF vs Control | female | hsa-miR-6511b-3p | 31.6 | -0.380 | 3.53E-02 | 4.62E-01 |
| HFrEF vs Control | female | hsa-miR-486-5p | 2175218.5 | -0.366 | 1.53E-02 | 4.62E-01 |
| HFrEF vs Control | female | hsa-miR-574-3p | 174.1 | 0.333 | 3.83E-02 | 4.62E-01 |
| HFrEF vs Control | female | hsa-miR-532-5p | 386.3 | 0.331 | 2.16E-02 | 4.62E-01 |
| HFrEF vs Control | female | hsa-miR-140-3p | 2748.3 | 0.323 | 2.06E-02 | 4.62E-01 |
| HFrEF vs Control | female | hsa-miR-423-5p | 17833.4 | -0.296 | 4.32E-02 | 4.95E-01 |
| HFrEF vs Control | female | hsa-miR-100-5p | 65.1 | 1.330 | 4.60E-02 | 4.98E-01 |
| HFrEF vs Control | female | hsa-miR-142-3p | 48.2 | -0.663 | 4.85E-02 | 4.98E-01 |
| HFrEF vs Control | female | hsa-miR-99a-5p | 8.3 | -0.533 | 5.72E-02 | 4.98E-01 |
| HFrEF vs Control | female | hsa-miR-29b-3p | 64.6 | 0.471 | 1.27E-01 | 4.98E-01 |
| HFrEF vs Control | female | hsa-miR-15b-3p | 195.3 | 0.470 | 7.15E-02 | 4.98E-01 |
| HFrEF vs Control | female | hsa-miR-15a-5p | 51.1 | 0.456 | 1.10E-01 | 4.98E-01 |
| HFrEF vs Control | female | hsa-miR-221-3p | 24.4 | 0.455 | 9.67E-02 | 4.98E-01 |
| HFrEF vs Control | female | hsa-miR-5010-3p | 11.0 | -0.449 | 6.50E-02 | 4.98E-01 |
| HFrEF vs Control | female | hsa-let-7e-5p | 12.5 | -0.448 | 9.32E-02 | 4.98E-01 |
| HFrEF vs Control | female | hsa-miR-7-5p | 117.4 | 0.445 | 1.09E-01 | 4.98E-01 |
| HFrEF vs Control | female | hsa-miR-4508 | 106.9 | -0.437 | 1.33E-01 | 4.98E-01 |
| HFrEF vs Control | female | hsa-miR-4466 | 9.2 | -0.435 | 8.94E-02 | 4.98E-01 |
| HFrEF vs Control | female | hsa-let-7d-3p | 151.3 | -0.422 | 6.70E-02 | 4.98E-01 |
| HFrEF vs Control | female | hsa-miR-1255b-5p | 25.9 | 0.420 | 1.19E-01 | 4.98E-01 |
| HFrEF vs Control | female | hsa-miR-6857-3p | 7.4 | -0.417 | 6.28E-02 | 4.98E-01 |
| HFrEF vs Control | female | hsa-miR-151a-3p | 817.0 | 0.417 | 6.37E-02 | 4.98E-01 |
| HFrEF vs Control | female | hsa-miR-3179 | 17.7 | 0.417 | 7.55E-02 | 4.98E-01 |
| HFrEF vs Control | female | hsa-miR-421 | 51.3 | 0.404 | 5.19E-02 | 4.98E-01 |
| HFrEF vs Control | female | hsa-miR-181a-3p | 9.8 | 0.361 | 1.19E-01 | 4.98E-01 |
| HFrEF vs Control | female | hsa-miR-30b-5p | 70.8 | 0.357 | 7.87E-02 | 4.98E-01 |
| HFrEF vs Control | female | hsa-miR-6877-5p | 9.6 | -0.348 | 9.35E-02 | 4.98E-01 |
| HFrEF vs Control | female | hsa-miR-106b-3p | 97.6 | 0.348 | 1.17E-01 | 4.98E-01 |
| HFrEF vs Control | female | hsa-miR-320b | 170.4 | -0.345 | 7.42E-02 | 4.98E-01 |
| HFrEF vs Control | female | hsa-miR-1843 | 22.0 | -0.344 | 1.32E-01 | 4.98E-01 |
| HFrEF vs Control | female | hsa-miR-1180-3p | 772.5 | -0.339 | 6.69E-02 | 4.98E-01 |
| HFrEF vs Control | female | hsa-miR-671-3p | 25.6 | -0.311 | 1.06E-01 | 4.98E-01 |
| HFrEF vs Control | female | hsa-miR-320d | 9.5 | -0.310 | 1.36E-01 | 4.98E-01 |
| HFrEF vs Control | female | hsa-miR-1260b | 184.1 | -0.309 | 1.41E-01 | 4.98E-01 |
| HFrEF vs Control | female | hsa-miR-186-5p | 220.9 | 0.306 | 5.57E-02 | 4.98E-01 |
| HFrEF vs Control | female | hsa-miR-191-5p | 290494.4 | 0.302 | 8.72E-02 | 4.98E-01 |
| HFrEF vs Control | female | hsa-miR-92b-3p | 631.1 | -0.294 | 1.35E-01 | 4.98E-01 |
| HFrEF vs Control | female | hsa-miR-6803-3p | 71.8 | -0.287 | 1.40E-01 | 4.98E-01 |
| HFrEF vs Control | female | hsa-miR-26a-5p | 928.1 | -0.286 | 1.22E-01 | 4.98E-01 |
| HFrEF vs Control | female | hsa-miR-194-5p | 28.3 | 0.272 | 1.18E-01 | 4.98E-01 |
| HFrEF vs Control | female | hsa-miR-3200-3p | 9.9 | 0.266 | 1.37E-01 | 4.98E-01 |
| HFrEF vs Control | female | hsa-miR-491-5p | 12.7 | -0.258 | 1.30E-01 | 4.98E-01 |
| HFrEF vs Control | female | hsa-miR-181b-5p | 32.5 | -0.248 | 1.20E-01 | 4.98E-01 |
| HFrEF vs Control | female | hsa-let-7c-5p | 89.6 | -0.248 | 1.34E-01 | 4.98E-01 |
| HFrEF vs Control | female | hsa-miR-30c-5p | 283.0 | 0.248 | 1.10E-01 | 4.98E-01 |
| HFrEF vs Control | female | hsa-miR-128-3p | 889.4 | 0.248 | 1.03E-01 | 4.98E-01 |
| HFrEF vs Control | female | hsa-miR-342-3p | 1930.9 | 0.243 | 1.40E-01 | 4.98E-01 |
| HFrEF vs Control | female | hsa-miR-3158-5p | 35.4 | 0.236 | 1.34E-01 | 4.98E-01 |
| HFrEF vs Control | female | hsa-miR-324-5p | 212.8 | 0.234 | 9.35E-02 | 4.98E-01 |
| HFrEF vs Control | female | hsa-miR-2110 | 851.7 | -0.228 | 7.73E-02 | 4.98E-01 |
| HFrEF vs Control | female | hsa-miR-1301-3p | 71.3 | -0.188 | 1.30E-01 | 4.98E-01 |
| HFrEF vs Control | female | hsa-miR-3940-3p | 202.4 | -0.333 | 1.48E-01 | 5.15E-01 |
| HFrEF vs Control | female | hsa-miR-21-5p | 14.7 | 0.406 | 1.52E-01 | 5.21E-01 |
| HFrEF vs Control | female | hsa-miR-185-5p | 22552.6 | 0.210 | 1.57E-01 | 5.29E-01 |
| HFrEF vs Control | female | hsa-miR-28-3p | 202.5 | 0.310 | 1.63E-01 | 5.41E-01 |
| HFrEF vs Control | female | hsa-miR-4659a-3p | 11.8 | -0.322 | 1.67E-01 | 5.46E-01 |
| HFrEF vs Control | female | hsa-miR-361-3p | 42.2 | 0.195 | 1.83E-01 | 5.90E-01 |
| HFrEF vs Control | female | hsa-miR-636 | 71.5 | -0.216 | 1.87E-01 | 5.93E-01 |
| HFrEF vs Control | female | hsa-miR-425-3p | 275.2 | 0.161 | 1.94E-01 | 6.08E-01 |
| HFrEF vs Control | female | hsa-miR-423-3p | 204.0 | -0.232 | 2.03E-01 | 6.28E-01 |
| HFrEF vs Control | female | hsa-miR-660-5p | 38.4 | 0.224 | 2.07E-01 | 6.32E-01 |
| HFrEF vs Control | female | hsa-miR-6750-5p | 9.1 | -0.281 | 2.11E-01 | 6.34E-01 |
| HFrEF vs Control | female | hsa-miR-122-5p | 16.3 | 0.535 | 2.21E-01 | 6.42E-01 |
| HFrEF vs Control | female | hsa-miR-3688-3p | 16.2 | 0.245 | 2.16E-01 | 6.42E-01 |
| HFrEF vs Control | female | hsa-miR-320a-3p | 12683.6 | -0.223 | 2.20E-01 | 6.42E-01 |
| HFrEF vs Control | female | hsa-miR-4685-3p | 33.8 | -0.282 | 2.26E-01 | 6.47E-01 |
| HFrEF vs Control | female | hsa-miR-4433b-5p | 24.6 | -0.305 | 2.30E-01 | 6.51E-01 |
| HFrEF vs Control | female | hsa-miR-15b-5p | 4479.3 | 0.322 | 2.45E-01 | 6.63E-01 |
| HFrEF vs Control | female | hsa-miR-145-5p | 17.7 | 0.290 | 2.45E-01 | 6.63E-01 |
| HFrEF vs Control | female | hsa-miR-576-5p | 139.2 | 0.273 | 2.46E-01 | 6.63E-01 |
| HFrEF vs Control | female | hsa-miR-30d-5p | 2292.1 | -0.149 | 2.39E-01 | 6.63E-01 |
| HFrEF vs Control | female | hsa-miR-10a-5p | 29.8 | 0.339 | 2.65E-01 | 6.66E-01 |
| HFrEF vs Control | female | hsa-miR-1287-5p | 12.7 | -0.317 | 2.60E-01 | 6.66E-01 |
| HFrEF vs Control | female | hsa-miR-3187-3p | 6.3 | -0.281 | 2.62E-01 | 6.66E-01 |
| HFrEF vs Control | female | hsa-miR-326 | 38.5 | -0.218 | 2.62E-01 | 6.66E-01 |
| HFrEF vs Control | female | hsa-miR-652-3p | 574.9 | -0.208 | 2.65E-01 | 6.66E-01 |
| HFrEF vs Control | female | hsa-miR-877-5p | 48.6 | -0.191 | 2.68E-01 | 6.66E-01 |
| HFrEF vs Control | female | hsa-miR-744-5p | 452.2 | -0.140 | 2.53E-01 | 6.66E-01 |
| HFrEF vs Control | female | hsa-miR-1285-3p | 13.2 | -0.253 | 2.82E-01 | 6.70E-01 |
| HFrEF vs Control | female | hsa-miR-324-3p | 32.3 | 0.209 | 2.85E-01 | 6.70E-01 |
| HFrEF vs Control | female | hsa-miR-197-3p | 184.7 | -0.205 | 2.85E-01 | 6.70E-01 |
| HFrEF vs Control | female | hsa-miR-3124-5p | 11.1 | 0.204 | 2.89E-01 | 6.70E-01 |
| HFrEF vs Control | female | hsa-miR-125a-5p | 220.1 | -0.196 | 2.76E-01 | 6.70E-01 |
| HFrEF vs Control | female | hsa-miR-1294 | 129.0 | 0.165 | 2.78E-01 | 6.70E-01 |
| HFrEF vs Control | female | hsa-miR-625-5p | 415.2 | 0.148 | 2.93E-01 | 6.70E-01 |
| HFrEF vs Control | female | hsa-miR-25-3p | 5366.3 | 0.129 | 2.90E-01 | 6.70E-01 |
| HFrEF vs Control | female | hsa-miR-941 | 232.3 | 0.232 | 3.05E-01 | 6.92E-01 |
| HFrEF vs Control | female | hsa-miR-378a-5p | 21.1 | 0.204 | 3.11E-01 | 6.99E-01 |
| HFrEF vs Control | female | hsa-miR-3667-5p | 16.2 | 0.290 | 3.18E-01 | 7.06E-01 |
| HFrEF vs Control | female | hsa-miR-103a-3p | 3977.0 | 0.164 | 3.25E-01 | 7.16E-01 |
| HFrEF vs Control | female | hsa-miR-331-3p | 24.1 | -0.181 | 3.29E-01 | 7.18E-01 |
| HFrEF vs Control | female | hsa-let-7i-3p | 12.0 | 0.184 | 3.38E-01 | 7.23E-01 |
| HFrEF vs Control | female | hsa-miR-328-3p | 44.8 | -0.178 | 3.35E-01 | 7.23E-01 |
| HFrEF vs Control | female | hsa-miR-130b-3p | 336.7 | 0.137 | 3.42E-01 | 7.24E-01 |
| HFrEF vs Control | female | hsa-miR-4723-5p | 9.2 | 0.193 | 3.63E-01 | 7.34E-01 |
| HFrEF vs Control | female | hsa-miR-17-3p | 89.3 | 0.192 | 3.70E-01 | 7.34E-01 |
| HFrEF vs Control | female | hsa-miR-1470 | 13.6 | -0.191 | 3.72E-01 | 7.34E-01 |
| HFrEF vs Control | female | hsa-miR-192-5p | 51.0 | 0.190 | 3.55E-01 | 7.34E-01 |
| HFrEF vs Control | female | hsa-miR-4732-3p | 1184.9 | 0.171 | 3.57E-01 | 7.34E-01 |
| HFrEF vs Control | female | hsa-miR-425-5p | 2011.2 | 0.162 | 3.56E-01 | 7.34E-01 |
| HFrEF vs Control | female | hsa-miR-378a-3p | 140.5 | 0.144 | 3.69E-01 | 7.34E-01 |
| HFrEF vs Control | female | hsa-miR-342-5p | 193.3 | -0.110 | 3.59E-01 | 7.34E-01 |
| HFrEF vs Control | female | hsa-miR-579-5p | 7.8 | 0.190 | 3.85E-01 | 7.48E-01 |
| HFrEF vs Control | female | hsa-miR-30e-5p | 134.6 | -0.153 | 3.85E-01 | 7.48E-01 |
| HFrEF vs Control | female | hsa-miR-1306-5p | 233.4 | 0.169 | 3.97E-01 | 7.65E-01 |
| HFrEF vs Control | female | hsa-miR-29c-3p | 11.9 | 0.211 | 4.01E-01 | 7.65E-01 |
| HFrEF vs Control | female | hsa-miR-6802-3p | 8.9 | -0.184 | 4.17E-01 | 7.70E-01 |
| HFrEF vs Control | female | hsa-miR-574-5p | 106.0 | -0.174 | 4.16E-01 | 7.70E-01 |
| HFrEF vs Control | female | hsa-miR-451a | 847.8 | 0.155 | 4.14E-01 | 7.70E-01 |
| HFrEF vs Control | female | hsa-miR-93-3p | 1043.0 | -0.129 | 4.14E-01 | 7.70E-01 |
| HFrEF vs Control | female | hsa-miR-4800-3p | 55.5 | -0.219 | 4.21E-01 | 7.71E-01 |
| HFrEF vs Control | female | hsa-miR-484 | 1600.2 | -0.169 | 4.36E-01 | 7.92E-01 |
| HFrEF vs Control | female | hsa-miR-1270 | 28.6 | 0.175 | 4.42E-01 | 7.97E-01 |
| HFrEF vs Control | female | hsa-miR-143-3p | 10.2 | 0.177 | 4.76E-01 | 8.07E-01 |
| HFrEF vs Control | female | hsa-miR-182-5p | 1249.0 | 0.177 | 4.74E-01 | 8.07E-01 |
| HFrEF vs Control | female | hsa-miR-4454 | 32.7 | 0.165 | 4.56E-01 | 8.07E-01 |
| HFrEF vs Control | female | hsa-miR-98-5p | 47.1 | 0.163 | 4.75E-01 | 8.07E-01 |
| HFrEF vs Control | female | hsa-miR-503-5p | 26.3 | 0.155 | 4.71E-01 | 8.07E-01 |
| HFrEF vs Control | female | hsa-miR-1229-3p | 10.5 | 0.143 | 4.69E-01 | 8.07E-01 |
| HFrEF vs Control | female | hsa-miR-345-5p | 41.0 | 0.123 | 4.83E-01 | 8.07E-01 |
| HFrEF vs Control | female | hsa-miR-3605-3p | 1000.7 | -0.118 | 4.62E-01 | 8.07E-01 |
| HFrEF vs Control | female | hsa-let-7b-3p | 21.7 | 0.118 | 4.83E-01 | 8.07E-01 |
| HFrEF vs Control | female | hsa-miR-6513-3p | 59.0 | 0.108 | 4.77E-01 | 8.07E-01 |
| HFrEF vs Control | female | hsa-miR-92b-5p | 16.9 | 0.131 | 4.91E-01 | 8.16E-01 |
| HFrEF vs Control | female | hsa-miR-4301 | 9.6 | 0.196 | 5.00E-01 | 8.19E-01 |
| HFrEF vs Control | female | hsa-miR-550a-3-5p | 36.6 | -0.098 | 4.98E-01 | 8.19E-01 |
| HFrEF vs Control | female | hsa-miR-339-5p | 324.4 | 0.129 | 5.07E-01 | 8.23E-01 |
| HFrEF vs Control | female | hsa-miR-17-5p | 13.1 | 0.166 | 5.14E-01 | 8.29E-01 |
| HFrEF vs Control | female | hsa-miR-181a-5p | 343.2 | -0.140 | 5.21E-01 | 8.29E-01 |
| HFrEF vs Control | female | hsa-miR-339-3p | 211.5 | 0.086 | 5.22E-01 | 8.29E-01 |
| HFrEF vs Control | female | hsa-miR-18b-3p | 9.0 | -0.161 | 5.56E-01 | 8.35E-01 |
| HFrEF vs Control | female | hsa-miR-664a-5p | 12.4 | 0.146 | 5.42E-01 | 8.35E-01 |
| HFrEF vs Control | female | hsa-miR-500a-3p | 10.1 | 0.142 | 5.44E-01 | 8.35E-01 |
| HFrEF vs Control | female | hsa-miR-10401-3p | 8.9 | 0.141 | 5.50E-01 | 8.35E-01 |
| HFrEF vs Control | female | hsa-miR-3200-5p | 19.0 | 0.139 | 5.42E-01 | 8.35E-01 |
| HFrEF vs Control | female | hsa-miR-500a-5p | 7.3 | -0.135 | 5.62E-01 | 8.35E-01 |
| HFrEF vs Control | female | hsa-miR-501-5p | 11.0 | 0.123 | 5.60E-01 | 8.35E-01 |
| HFrEF vs Control | female | hsa-let-7f-5p | 3153.9 | 0.121 | 5.41E-01 | 8.35E-01 |
| HFrEF vs Control | female | hsa-miR-4448 | 13.3 | -0.111 | 5.31E-01 | 8.35E-01 |
| HFrEF vs Control | female | hsa-miR-1226-3p | 113.9 | 0.092 | 5.53E-01 | 8.35E-01 |
| HFrEF vs Control | female | hsa-miR-18a-3p | 13.4 | 0.131 | 5.68E-01 | 8.37E-01 |
| HFrEF vs Control | female | hsa-miR-628-3p | 13.5 | 0.127 | 5.70E-01 | 8.37E-01 |
| HFrEF vs Control | female | hsa-miR-505-3p | 15.0 | 0.100 | 5.77E-01 | 8.42E-01 |
| HFrEF vs Control | female | hsa-miR-126-5p | 32.7 | -0.230 | 5.84E-01 | 8.47E-01 |
| HFrEF vs Control | female | hsa-miR-361-5p | 198.4 | -0.079 | 5.92E-01 | 8.53E-01 |
| HFrEF vs Control | female | hsa-miR-191-3p | 24.3 | 0.085 | 6.00E-01 | 8.53E-01 |
| HFrEF vs Control | female | hsa-miR-24-3p | 285.9 | 0.061 | 5.97E-01 | 8.53E-01 |
| HFrEF vs Control | female | hsa-miR-664a-3p | 8.1 | 0.137 | 6.04E-01 | 8.54E-01 |
| HFrEF vs Control | female | hsa-miR-20a-5p | 56.0 | -0.136 | 6.30E-01 | 8.60E-01 |
| HFrEF vs Control | female | hsa-miR-409-3p | 72.8 | -0.132 | 6.41E-01 | 8.60E-01 |
| HFrEF vs Control | female | hsa-miR-3135b | 15.4 | 0.119 | 6.27E-01 | 8.60E-01 |
| HFrEF vs Control | female | hsa-miR-16-5p | 6087.5 | 0.102 | 6.30E-01 | 8.60E-01 |
| HFrEF vs Control | female | hsa-miR-93-5p | 9091.4 | 0.098 | 6.41E-01 | 8.60E-01 |
| HFrEF vs Control | female | hsa-miR-11401 | 8.4 | -0.096 | 6.49E-01 | 8.60E-01 |
| HFrEF vs Control | female | hsa-let-7g-5p | 5448.4 | 0.095 | 6.42E-01 | 8.60E-01 |
| HFrEF vs Control | female | hsa-miR-1307-3p | 72.8 | -0.091 | 6.20E-01 | 8.60E-01 |
| HFrEF vs Control | female | hsa-miR-23b-3p | 27.9 | 0.086 | 6.54E-01 | 8.60E-01 |
| HFrEF vs Control | female | hsa-miR-629-3p | 23.0 | 0.082 | 6.51E-01 | 8.60E-01 |
| HFrEF vs Control | female | hsa-miR-6511a-3p | 82.2 | 0.077 | 6.40E-01 | 8.60E-01 |
| HFrEF vs Control | female | hsa-miR-505-5p | 456.2 | 0.068 | 6.45E-01 | 8.60E-01 |
| HFrEF vs Control | female | hsa-miR-20b-5p | 205.3 | 0.104 | 6.62E-01 | 8.66E-01 |
| HFrEF vs Control | female | hsa-miR-25-5p | 56.5 | -0.072 | 6.75E-01 | 8.75E-01 |
| HFrEF vs Control | female | hsa-miR-629-5p | 427.0 | 0.057 | 6.76E-01 | 8.75E-01 |
| HFrEF vs Control | female | hsa-miR-4732-5p | 310.4 | 0.054 | 6.82E-01 | 8.78E-01 |
| HFrEF vs Control | female | hsa-miR-106b-5p | 13.8 | 0.109 | 6.92E-01 | 8.78E-01 |
| HFrEF vs Control | female | hsa-miR-26b-5p | 538.8 | -0.092 | 6.98E-01 | 8.78E-01 |
| HFrEF vs Control | female | hsa-let-7b-5p | 51072.8 | -0.079 | 6.96E-01 | 8.78E-01 |
| HFrEF vs Control | female | hsa-miR-532-3p | 1372.3 | 0.060 | 6.92E-01 | 8.78E-01 |
| HFrEF vs Control | female | hsa-miR-183-5p | 62.4 | -0.081 | 7.02E-01 | 8.79E-01 |
| HFrEF vs Control | female | hsa-miR-6820-3p | 13.3 | -0.097 | 7.06E-01 | 8.79E-01 |
| HFrEF vs Control | female | hsa-miR-1976 | 13.3 | 0.077 | 7.11E-01 | 8.80E-01 |
| HFrEF vs Control | female | hsa-miR-99b-5p | 145.9 | -0.081 | 7.21E-01 | 8.84E-01 |
| HFrEF vs Control | female | hsa-miR-7706 | 49.8 | -0.057 | 7.22E-01 | 8.84E-01 |
| HFrEF vs Control | female | hsa-miR-30e-3p | 445.0 | 0.082 | 7.31E-01 | 8.86E-01 |
| HFrEF vs Control | female | hsa-miR-501-3p | 26.7 | -0.063 | 7.31E-01 | 8.86E-01 |
| HFrEF vs Control | female | hsa-miR-1304-3p | 72.0 | 0.045 | 7.40E-01 | 8.91E-01 |
| HFrEF vs Control | female | hsa-miR-5189-3p | 33.0 | -0.172 | 7.86E-01 | 9.01E-01 |
| HFrEF vs Control | female | hsa-miR-323b-3p | 9.5 | -0.108 | 7.88E-01 | 9.01E-01 |
| HFrEF vs Control | female | hsa-miR-3074-5p | 14.8 | -0.086 | 8.09E-01 | 9.01E-01 |
| HFrEF vs Control | female | hsa-miR-627-5p | 9.7 | -0.078 | 7.90E-01 | 9.01E-01 |
| HFrEF vs Control | female | hsa-miR-3173-5p | 10.7 | -0.066 | 7.89E-01 | 9.01E-01 |
| HFrEF vs Control | female | hsa-miR-363-3p | 24.6 | 0.060 | 8.14E-01 | 9.01E-01 |
| HFrEF vs Control | female | hsa-miR-5010-5p | 22.1 | 0.058 | 7.92E-01 | 9.01E-01 |
| HFrEF vs Control | female | hsa-miR-22-3p | 728.3 | 0.050 | 7.78E-01 | 9.01E-01 |
| HFrEF vs Control | female | hsa-miR-942-5p | 524.7 | 0.049 | 7.76E-01 | 9.01E-01 |
| HFrEF vs Control | female | hsa-miR-362-5p | 47.7 | 0.048 | 7.60E-01 | 9.01E-01 |
| HFrEF vs Control | female | hsa-miR-107 | 900.0 | -0.048 | 8.07E-01 | 9.01E-01 |
| HFrEF vs Control | female | hsa-miR-92a-3p | 17772.5 | -0.047 | 7.95E-01 | 9.01E-01 |
| HFrEF vs Control | female | hsa-miR-151a-5p | 10041.8 | 0.044 | 7.99E-01 | 9.01E-01 |
| HFrEF vs Control | female | hsa-miR-155-5p | 52.6 | -0.042 | 7.93E-01 | 9.01E-01 |
| HFrEF vs Control | female | hsa-let-7d-5p | 6246.7 | -0.042 | 8.00E-01 | 9.01E-01 |
| HFrEF vs Control | female | hsa-miR-424-3p | 160.5 | -0.038 | 8.14E-01 | 9.01E-01 |
| HFrEF vs Control | female | hsa-miR-181a-2-3p | 305.6 | -0.028 | 7.75E-01 | 9.01E-01 |
| HFrEF vs Control | female | hsa-miR-1908-5p | 12.7 | 0.059 | 8.22E-01 | 9.05E-01 |
| HFrEF vs Control | female | hsa-miR-126-3p | 403.8 | 0.062 | 8.34E-01 | 9.07E-01 |
| HFrEF vs Control | female | hsa-miR-151b | 35.9 | -0.045 | 8.36E-01 | 9.07E-01 |
| HFrEF vs Control | female | hsa-miR-937-3p | 32.6 | -0.035 | 8.31E-01 | 9.07E-01 |
| HFrEF vs Control | female | hsa-miR-7977 | 71.4 | -0.032 | 8.43E-01 | 9.11E-01 |
| HFrEF vs Control | female | hsa-miR-183-3p | 101.5 | -0.034 | 8.53E-01 | 9.17E-01 |
| HFrEF vs Control | female | hsa-miR-29a-3p | 101.5 | 0.029 | 8.79E-01 | 9.41E-01 |
| HFrEF vs Control | female | hsa-miR-10527-5p | 6.2 | 0.032 | 8.95E-01 | 9.51E-01 |
| HFrEF vs Control | female | hsa-miR-3157-5p | 11.4 | -0.027 | 9.02E-01 | 9.51E-01 |
| HFrEF vs Control | female | hsa-miR-150-5p | 1548.7 | -0.025 | 8.97E-01 | 9.51E-01 |
| HFrEF vs Control | female | hsa-miR-30c-1-3p | 41.7 | -0.021 | 9.05E-01 | 9.51E-01 |
| HFrEF vs Control | female | hsa-miR-130b-5p | 13.4 | -0.025 | 9.15E-01 | 9.53E-01 |
| HFrEF vs Control | female | hsa-miR-589-5p | 137.8 | -0.013 | 9.14E-01 | 9.53E-01 |
| HFrEF vs Control | female | hsa-let-7i-5p | 11291.8 | -0.017 | 9.41E-01 | 9.66E-01 |
| HFrEF vs Control | female | hsa-miR-130a-3p | 618.7 | -0.015 | 9.40E-01 | 9.66E-01 |
| HFrEF vs Control | female | hsa-let-7a-5p | 29752.7 | -0.013 | 9.38E-01 | 9.66E-01 |
| HFrEF vs Control | female | hsa-miR-3928-3p | 10.2 | -0.012 | 9.49E-01 | 9.70E-01 |
| HFrEF vs Control | female | hsa-miR-550a-5p | 79.2 | 0.008 | 9.57E-01 | 9.74E-01 |
| HFrEF vs Control | female | hsa-miR-6734-5p | 18.2 | 0.008 | 9.70E-01 | 9.82E-01 |
| HFrEF vs Control | female | hsa-miR-200c-3p | 7.7 | -0.002 | 9.93E-01 | 9.95E-01 |
| HFrEF vs Control | female | hsa-miR-23a-3p | 92.0 | -0.002 | 9.92E-01 | 9.95E-01 |
| HFrEF vs Control | female | hsa-miR-1292-5p | 53.5 | 0.001 | 9.95E-01 | 9.95E-01 |
| HFrEF vs Control | male | hsa-miR-30b-5p | 74.1 | 0.858 | 1.90E-07 | 3.22E-05 |
| HFrEF vs Control | male | hsa-miR-128-3p | 974.8 | 0.576 | 2.81E-07 | 3.22E-05 |
| HFrEF vs Control | male | hsa-miR-192-5p | 57.6 | 0.721 | 6.62E-07 | 5.05E-05 |
| HFrEF vs Control | male | hsa-miR-6803-3p | 84.5 | -0.691 | 1.65E-06 | 7.58E-05 |
| HFrEF vs Control | male | hsa-miR-30c-5p | 301.9 | 0.603 | 1.46E-06 | 7.58E-05 |
| HFrEF vs Control | male | hsa-miR-223-3p | 2984.4 | 0.611 | 3.98E-06 | 1.52E-04 |
| HFrEF vs Control | male | hsa-miR-4508 | 134.0 | -0.946 | 6.11E-06 | 1.72E-04 |
| HFrEF vs Control | male | hsa-miR-142-5p | 3378.5 | 0.739 | 6.51E-06 | 1.72E-04 |
| HFrEF vs Control | male | hsa-miR-186-5p | 263.1 | 0.494 | 6.76E-06 | 1.72E-04 |
| HFrEF vs Control | male | hsa-miR-1260b | 214.8 | -0.575 | 1.60E-05 | 3.67E-04 |
| HFrEF vs Control | male | hsa-miR-15b-3p | 203.7 | 0.823 | 1.97E-05 | 4.11E-04 |
| HFrEF vs Control | male | hsa-miR-151a-3p | 882.3 | 0.552 | 2.79E-05 | 5.32E-04 |
| HFrEF vs Control | male | hsa-miR-29b-3p | 70.4 | 0.868 | 4.14E-05 | 7.29E-04 |
| HFrEF vs Control | male | hsa-miR-335-5p | 65.8 | 0.824 | 5.06E-05 | 8.27E-04 |
| HFrEF vs Control | male | hsa-miR-3135b | 15.6 | -0.731 | 8.31E-05 | 1.27E-03 |
| HFrEF vs Control | male | hsa-let-7c-5p | 119.4 | -0.563 | 9.65E-05 | 1.38E-03 |
| HFrEF vs Control | male | hsa-miR-28-3p | 205.4 | 0.585 | 1.15E-04 | 1.55E-03 |
| HFrEF vs Control | male | hsa-miR-652-3p | 681.8 | -0.402 | 1.33E-04 | 1.70E-03 |
| HFrEF vs Control | male | hsa-miR-361-3p | 45.9 | 0.396 | 1.87E-04 | 2.26E-03 |
| HFrEF vs Control | male | hsa-miR-194-5p | 34.1 | 0.629 | 2.18E-04 | 2.50E-03 |
| HFrEF vs Control | male | hsa-miR-142-3p | 40.5 | 0.807 | 2.42E-04 | 2.64E-03 |
| HFrEF vs Control | male | hsa-miR-942-5p | 621.4 | 0.443 | 2.84E-04 | 2.95E-03 |
| HFrEF vs Control | male | hsa-let-7i-5p | 14803.8 | -0.579 | 3.59E-04 | 3.58E-03 |
| HFrEF vs Control | male | hsa-miR-23a-3p | 107.2 | 0.516 | 4.23E-04 | 4.04E-03 |
| HFrEF vs Control | male | hsa-miR-1301-3p | 88.2 | -0.335 | 4.98E-04 | 4.57E-03 |
| HFrEF vs Control | male | hsa-miR-342-3p | 2161.9 | 0.408 | 5.74E-04 | 5.05E-03 |
| HFrEF vs Control | male | hsa-miR-1287-5p | 18.6 | -0.687 | 7.23E-04 | 6.11E-03 |
| HFrEF vs Control | male | hsa-miR-451a | 978.9 | 0.532 | 7.74E-04 | 6.11E-03 |
| HFrEF vs Control | male | hsa-miR-93-5p | 11834.5 | -0.531 | 7.68E-04 | 6.11E-03 |
| HFrEF vs Control | male | hsa-miR-181a-5p | 419.0 | -0.446 | 8.69E-04 | 6.63E-03 |
| HFrEF vs Control | male | hsa-miR-182-5p | 1460.7 | 0.632 | 9.29E-04 | 6.86E-03 |
| HFrEF vs Control | male | hsa-miR-324-5p | 243.1 | 0.356 | 1.20E-03 | 8.58E-03 |
| HFrEF vs Control | male | hsa-miR-3200-3p | 12.1 | 0.483 | 1.35E-03 | 9.36E-03 |
| HFrEF vs Control | male | hsa-miR-22-3p | 835.4 | 0.367 | 1.73E-03 | 1.16E-02 |
| HFrEF vs Control | male | hsa-miR-10a-5p | 33.0 | 0.831 | 2.26E-03 | 1.48E-02 |
| HFrEF vs Control | male | hsa-let-7b-5p | 70566.3 | -0.518 | 2.76E-03 | 1.71E-02 |
| HFrEF vs Control | male | hsa-miR-505-3p | 16.6 | 0.400 | 2.74E-03 | 1.71E-02 |
| HFrEF vs Control | male | hsa-miR-1843 | 26.8 | -0.385 | 3.22E-03 | 1.89E-02 |
| HFrEF vs Control | male | hsa-miR-424-3p | 194.0 | -0.346 | 3.16E-03 | 1.89E-02 |
| HFrEF vs Control | male | hsa-miR-576-5p | 159.4 | 0.562 | 3.37E-03 | 1.93E-02 |
| HFrEF vs Control | male | hsa-miR-421 | 56.1 | 0.421 | 3.52E-03 | 1.96E-02 |
| HFrEF vs Control | male | hsa-miR-193a-5p | 76.7 | -0.439 | 4.00E-03 | 2.18E-02 |
| HFrEF vs Control | male | hsa-miR-1294 | 162.1 | -0.334 | 4.48E-03 | 2.38E-02 |
| HFrEF vs Control | male | hsa-miR-30e-5p | 146.2 | -0.313 | 5.30E-03 | 2.69E-02 |
| HFrEF vs Control | male | hsa-miR-25-3p | 6395.6 | 0.259 | 5.26E-03 | 2.69E-02 |
| HFrEF vs Control | male | hsa-miR-7706 | 57.0 | -0.345 | 5.76E-03 | 2.87E-02 |
| HFrEF vs Control | male | hsa-miR-181a-3p | 10.0 | 0.455 | 6.41E-03 | 3.02E-02 |
| HFrEF vs Control | male | hsa-miR-107 | 1166.1 | -0.397 | 6.36E-03 | 3.02E-02 |
| HFrEF vs Control | male | hsa-miR-4448 | 15.0 | -0.379 | 6.72E-03 | 3.02E-02 |
| HFrEF vs Control | male | hsa-miR-140-3p | 3173.3 | 0.332 | 6.47E-03 | 3.02E-02 |
| HFrEF vs Control | male | hsa-miR-181a-2-3p | 334.3 | 0.210 | 6.72E-03 | 3.02E-02 |
| HFrEF vs Control | male | hsa-miR-1306-5p | 254.9 | 0.390 | 6.87E-03 | 3.02E-02 |
| HFrEF vs Control | male | hsa-miR-320a-3p | 15849.0 | -0.344 | 8.08E-03 | 3.49E-02 |
| HFrEF vs Control | male | hsa-miR-5010-3p | 11.6 | -0.437 | 9.72E-03 | 4.12E-02 |
| HFrEF vs Control | male | hsa-let-7a-5p | 37077.9 | -0.321 | 9.98E-03 | 4.16E-02 |
| HFrEF vs Control | male | hsa-miR-339-5p | 378.6 | 0.375 | 1.03E-02 | 4.20E-02 |
| HFrEF vs Control | male | hsa-miR-425-5p | 2313.7 | 0.315 | 1.06E-02 | 4.26E-02 |
| HFrEF vs Control | male | hsa-miR-3615 | 2224.4 | -0.445 | 1.12E-02 | 4.34E-02 |
| HFrEF vs Control | male | hsa-let-7d-5p | 7850.8 | -0.281 | 1.11E-02 | 4.34E-02 |
| HFrEF vs Control | male | hsa-miR-7-5p | 127.0 | 0.416 | 1.51E-02 | 5.75E-02 |
| HFrEF vs Control | male | hsa-miR-6513-3p | 70.0 | 0.264 | 1.68E-02 | 6.30E-02 |
| HFrEF vs Control | male | hsa-miR-20a-5p | 64.3 | -0.498 | 1.87E-02 | 6.46E-02 |
| HFrEF vs Control | male | hsa-miR-3173-5p | 13.9 | -0.415 | 1.78E-02 | 6.46E-02 |
| HFrEF vs Control | male | hsa-miR-29a-3p | 122.3 | 0.371 | 1.89E-02 | 6.46E-02 |
| HFrEF vs Control | male | hsa-miR-1260a | 33.6 | -0.367 | 1.84E-02 | 6.46E-02 |
| HFrEF vs Control | male | hsa-miR-320b | 209.7 | -0.329 | 1.81E-02 | 6.46E-02 |
| HFrEF vs Control | male | hsa-let-7b-3p | 23.0 | 0.292 | 1.79E-02 | 6.46E-02 |
| HFrEF vs Control | male | hsa-miR-4685-3p | 39.2 | -0.399 | 2.17E-02 | 7.29E-02 |
| HFrEF vs Control | male | hsa-miR-1275 | 14.3 | -0.406 | 2.26E-02 | 7.49E-02 |
| HFrEF vs Control | male | hsa-miR-4732-3p | 1212.4 | 0.306 | 2.33E-02 | 7.63E-02 |
| HFrEF vs Control | male | hsa-miR-744-5p | 546.6 | -0.220 | 2.39E-02 | 7.70E-02 |
| HFrEF vs Control | male | hsa-miR-627-5p | 11.2 | 0.504 | 2.47E-02 | 7.72E-02 |
| HFrEF vs Control | male | hsa-miR-629-5p | 539.6 | -0.247 | 2.44E-02 | 7.72E-02 |
| HFrEF vs Control | male | hsa-miR-342-5p | 231.4 | -0.226 | 2.50E-02 | 7.72E-02 |
| HFrEF vs Control | male | hsa-miR-574-5p | 153.6 | -0.422 | 2.85E-02 | 8.40E-02 |
| HFrEF vs Control | male | hsa-miR-345-5p | 48.1 | 0.250 | 2.86E-02 | 8.40E-02 |
| HFrEF vs Control | male | hsa-miR-4732-5p | 357.2 | -0.246 | 2.85E-02 | 8.40E-02 |
| HFrEF vs Control | male | hsa-miR-191-5p | 339789.3 | 0.245 | 2.85E-02 | 8.40E-02 |
| HFrEF vs Control | male | hsa-miR-122-5p | 22.3 | -0.646 | 3.04E-02 | 8.81E-02 |
| HFrEF vs Control | male | hsa-miR-574-3p | 189.6 | 0.273 | 3.15E-02 | 9.03E-02 |
| HFrEF vs Control | male | hsa-miR-125a-5p | 244.8 | 0.284 | 3.38E-02 | 9.55E-02 |
| HFrEF vs Control | male | hsa-miR-378a-5p | 24.5 | 0.284 | 3.45E-02 | 9.63E-02 |
| HFrEF vs Control | male | hsa-miR-320c | 39.9 | -0.351 | 3.53E-02 | 9.74E-02 |
| HFrEF vs Control | male | hsa-miR-150-5p | 1778.9 | 0.316 | 3.90E-02 | 1.06E-01 |
| HFrEF vs Control | male | hsa-miR-4800-3p | 64.1 | -0.386 | 4.00E-02 | 1.08E-01 |
| HFrEF vs Control | male | hsa-miR-1307-3p | 91.9 | -0.286 | 4.09E-02 | 1.08E-01 |
| HFrEF vs Control | male | hsa-miR-3688-3p | 17.4 | 0.283 | 4.08E-02 | 1.08E-01 |
| HFrEF vs Control | male | hsa-miR-181b-5p | 41.3 | -0.241 | 4.26E-02 | 1.11E-01 |
| HFrEF vs Control | male | hsa-miR-18a-3p | 13.7 | 0.352 | 4.36E-02 | 1.12E-01 |
| HFrEF vs Control | male | hsa-miR-23b-3p | 30.4 | 0.266 | 4.39E-02 | 1.12E-01 |
| HFrEF vs Control | male | hsa-miR-106b-5p | 16.4 | -0.315 | 4.62E-02 | 1.16E-01 |
| HFrEF vs Control | male | hsa-miR-4466 | 10.2 | -0.405 | 4.68E-02 | 1.16E-01 |
| HFrEF vs Control | male | hsa-miR-4433b-5p | 24.3 | -0.334 | 5.02E-02 | 1.20E-01 |
| HFrEF vs Control | male | hsa-let-7g-5p | 6801.3 | -0.320 | 4.98E-02 | 1.20E-01 |
| HFrEF vs Control | male | hsa-miR-25-5p | 73.4 | -0.282 | 4.95E-02 | 1.20E-01 |
| HFrEF vs Control | male | hsa-miR-1180-3p | 1007.6 | -0.262 | 4.98E-02 | 1.20E-01 |
| HFrEF vs Control | male | hsa-miR-324-3p | 32.8 | 0.234 | 5.12E-02 | 1.21E-01 |
| HFrEF vs Control | male | hsa-miR-4454 | 37.3 | 0.277 | 5.74E-02 | 1.34E-01 |
| HFrEF vs Control | male | hsa-miR-579-5p | 10.4 | -0.309 | 5.87E-02 | 1.36E-01 |
| HFrEF vs Control | male | hsa-miR-200c-3p | 10.8 | -0.286 | 6.09E-02 | 1.40E-01 |
| HFrEF vs Control | male | hsa-miR-500a-5p | 10.3 | 0.283 | 6.31E-02 | 1.43E-01 |
| HFrEF vs Control | male | hsa-miR-2110 | 1094.1 | -0.232 | 6.39E-02 | 1.44E-01 |
| HFrEF vs Control | male | hsa-miR-6857-3p | 6.6 | 0.296 | 6.49E-02 | 1.44E-01 |
| HFrEF vs Control | male | hsa-miR-339-3p | 253.0 | 0.165 | 7.15E-02 | 1.57E-01 |
| HFrEF vs Control | male | hsa-miR-6734-5p | 22.0 | -0.309 | 7.32E-02 | 1.57E-01 |
| HFrEF vs Control | male | hsa-miR-550a-3-5p | 42.5 | -0.209 | 7.35E-02 | 1.57E-01 |
| HFrEF vs Control | male | hsa-miR-93-3p | 1175.8 | 0.201 | 7.29E-02 | 1.57E-01 |
| HFrEF vs Control | male | hsa-miR-103a-3p | 4997.6 | -0.224 | 7.77E-02 | 1.65E-01 |
| HFrEF vs Control | male | hsa-miR-17-3p | 101.5 | -0.239 | 7.95E-02 | 1.67E-01 |
| HFrEF vs Control | male | hsa-miR-3158-5p | 36.6 | 0.219 | 8.08E-02 | 1.68E-01 |
| HFrEF vs Control | male | hsa-miR-6511b-3p | 38.0 | -0.243 | 8.27E-02 | 1.71E-01 |
| HFrEF vs Control | male | hsa-miR-99a-5p | 10.5 | 0.344 | 8.48E-02 | 1.73E-01 |
| HFrEF vs Control | male | hsa-miR-486-5p | 2545093.2 | -0.224 | 8.60E-02 | 1.74E-01 |
| HFrEF vs Control | male | hsa-miR-20b-5p | 250.6 | -0.309 | 9.61E-02 | 1.92E-01 |
| HFrEF vs Control | male | hsa-miR-409-3p | 78.8 | 0.299 | 9.65E-02 | 1.92E-01 |
| HFrEF vs Control | male | hsa-miR-26b-5p | 669.3 | -0.289 | 1.03E-01 | 2.01E-01 |
| HFrEF vs Control | male | hsa-miR-484 | 1815.7 | -0.263 | 1.03E-01 | 2.01E-01 |
| HFrEF vs Control | male | hsa-miR-3179 | 23.4 | 0.253 | 1.04E-01 | 2.01E-01 |
| HFrEF vs Control | male | hsa-miR-30d-5p | 2738.2 | -0.147 | 1.06E-01 | 2.04E-01 |
| HFrEF vs Control | male | hsa-let-7e-5p | 18.8 | -0.255 | 1.11E-01 | 2.11E-01 |
| HFrEF vs Control | male | hsa-miR-532-3p | 1542.0 | 0.174 | 1.14E-01 | 2.16E-01 |
| HFrEF vs Control | male | hsa-miR-323b-3p | 10.1 | 0.391 | 1.20E-01 | 2.26E-01 |
| HFrEF vs Control | male | hsa-miR-29c-3p | 14.5 | 0.294 | 1.25E-01 | 2.32E-01 |
| HFrEF vs Control | male | hsa-miR-1470 | 13.0 | 0.262 | 1.25E-01 | 2.32E-01 |
| HFrEF vs Control | male | hsa-miR-361-5p | 216.8 | 0.167 | 1.32E-01 | 2.42E-01 |
| HFrEF vs Control | male | hsa-miR-185-5p | 26155.9 | 0.140 | 1.38E-01 | 2.50E-01 |
| HFrEF vs Control | male | hsa-let-7d-3p | 146.7 | 0.205 | 1.40E-01 | 2.52E-01 |
| HFrEF vs Control | male | hsa-miR-1255b-5p | 31.6 | 0.226 | 1.41E-01 | 2.52E-01 |
| HFrEF vs Control | male | hsa-miR-363-3p | 29.3 | 0.288 | 1.47E-01 | 2.60E-01 |
| HFrEF vs Control | male | hsa-miR-629-3p | 26.5 | 0.176 | 1.56E-01 | 2.75E-01 |
| HFrEF vs Control | male | hsa-miR-671-3p | 31.9 | 0.190 | 1.61E-01 | 2.79E-01 |
| HFrEF vs Control | male | hsa-miR-331-3p | 27.7 | -0.182 | 1.61E-01 | 2.79E-01 |
| HFrEF vs Control | male | hsa-miR-99b-5p | 164.4 | 0.228 | 1.67E-01 | 2.88E-01 |
| HFrEF vs Control | male | hsa-miR-1285-3p | 16.6 | 0.197 | 1.78E-01 | 3.03E-01 |
| HFrEF vs Control | male | hsa-miR-877-5p | 59.9 | -0.186 | 1.82E-01 | 3.07E-01 |
| HFrEF vs Control | male | hsa-miR-378a-3p | 172.3 | -0.146 | 1.82E-01 | 3.07E-01 |
| HFrEF vs Control | male | hsa-miR-3124-5p | 15.7 | -0.216 | 1.86E-01 | 3.11E-01 |
| HFrEF vs Control | male | hsa-miR-1976 | 14.8 | -0.228 | 1.94E-01 | 3.22E-01 |
| HFrEF vs Control | male | hsa-miR-106b-3p | 104.7 | 0.193 | 2.01E-01 | 3.31E-01 |
| HFrEF vs Control | male | hsa-miR-3928-3p | 13.0 | -0.197 | 2.09E-01 | 3.42E-01 |
| HFrEF vs Control | male | hsa-miR-3074-5p | 14.5 | -0.261 | 2.33E-01 | 3.78E-01 |
| HFrEF vs Control | male | hsa-miR-320d | 10.8 | -0.171 | 2.42E-01 | 3.90E-01 |
| HFrEF vs Control | male | hsa-miR-6750-5p | 12.0 | -0.182 | 2.48E-01 | 3.98E-01 |
| HFrEF vs Control | male | hsa-miR-15a-5p | 63.9 | 0.277 | 2.53E-01 | 4.03E-01 |
| HFrEF vs Control | male | hsa-miR-16-5p | 7391.4 | 0.199 | 2.63E-01 | 4.15E-01 |
| HFrEF vs Control | male | hsa-miR-221-3p | 27.6 | 0.229 | 2.77E-01 | 4.35E-01 |
| HFrEF vs Control | male | hsa-miR-17-5p | 14.6 | -0.208 | 2.90E-01 | 4.52E-01 |
| HFrEF vs Control | male | hsa-miR-3157-5p | 14.0 | -0.171 | 2.98E-01 | 4.61E-01 |
| HFrEF vs Control | male | hsa-miR-143-3p | 11.1 | 0.172 | 3.08E-01 | 4.73E-01 |
| HFrEF vs Control | male | hsa-miR-4659a-3p | 14.5 | 0.165 | 3.13E-01 | 4.77E-01 |
| HFrEF vs Control | male | hsa-miR-7977 | 81.8 | 0.108 | 3.31E-01 | 5.01E-01 |
| HFrEF vs Control | male | hsa-miR-500a-3p | 12.5 | 0.149 | 3.42E-01 | 5.05E-01 |
| HFrEF vs Control | male | hsa-miR-10527-5p | 9.7 | -0.144 | 3.42E-01 | 5.05E-01 |
| HFrEF vs Control | male | hsa-miR-423-5p | 21609.6 | -0.125 | 3.36E-01 | 5.05E-01 |
| HFrEF vs Control | male | hsa-miR-1292-5p | 66.7 | -0.115 | 3.39E-01 | 5.05E-01 |
| HFrEF vs Control | male | hsa-miR-18b-3p | 9.7 | -0.175 | 3.62E-01 | 5.31E-01 |
| HFrEF vs Control | male | hsa-miR-4301 | 10.6 | -0.197 | 3.65E-01 | 5.33E-01 |
| HFrEF vs Control | male | hsa-miR-5189-3p | 36.4 | -0.459 | 3.88E-01 | 5.55E-01 |
| HFrEF vs Control | male | hsa-miR-126-3p | 471.7 | -0.213 | 3.86E-01 | 5.55E-01 |
| HFrEF vs Control | male | hsa-miR-491-5p | 15.0 | -0.115 | 3.83E-01 | 5.55E-01 |
| HFrEF vs Control | male | hsa-miR-1226-3p | 136.1 | 0.105 | 4.03E-01 | 5.73E-01 |
| HFrEF vs Control | male | hsa-miR-11401 | 9.5 | -0.166 | 4.14E-01 | 5.85E-01 |
| HFrEF vs Control | male | hsa-miR-503-5p | 34.7 | -0.125 | 4.27E-01 | 6.00E-01 |
| HFrEF vs Control | male | hsa-miR-6802-3p | 9.5 | -0.123 | 4.31E-01 | 6.02E-01 |
| HFrEF vs Control | male | hsa-miR-98-5p | 56.8 | 0.142 | 4.36E-01 | 6.06E-01 |
| HFrEF vs Control | male | hsa-miR-4723-5p | 10.4 | -0.127 | 4.43E-01 | 6.10E-01 |
| HFrEF vs Control | male | hsa-miR-15b-5p | 5371.0 | 0.157 | 4.54E-01 | 6.20E-01 |
| HFrEF vs Control | male | hsa-miR-550a-5p | 83.9 | 0.077 | 4.55E-01 | 6.20E-01 |
| HFrEF vs Control | male | hsa-miR-532-5p | 466.1 | 0.090 | 4.63E-01 | 6.23E-01 |
| HFrEF vs Control | male | hsa-miR-425-3p | 318.0 | -0.078 | 4.61E-01 | 6.23E-01 |
| HFrEF vs Control | male | hsa-miR-625-5p | 477.0 | 0.097 | 4.70E-01 | 6.30E-01 |
| HFrEF vs Control | male | hsa-miR-210-3p | 229.8 | -0.107 | 4.84E-01 | 6.44E-01 |
| HFrEF vs Control | male | hsa-miR-22-5p | 15.3 | 0.110 | 4.87E-01 | 6.44E-01 |
| HFrEF vs Control | male | hsa-miR-937-3p | 38.2 | 0.088 | 4.90E-01 | 6.45E-01 |
| HFrEF vs Control | male | hsa-miR-6820-3p | 14.4 | 0.107 | 4.99E-01 | 6.49E-01 |
| HFrEF vs Control | male | hsa-miR-664a-5p | 16.6 | -0.103 | 4.97E-01 | 6.49E-01 |
| HFrEF vs Control | male | hsa-miR-423-3p | 221.4 | 0.092 | 5.03E-01 | 6.51E-01 |
| HFrEF vs Control | male | hsa-miR-3187-3p | 9.2 | -0.119 | 5.16E-01 | 6.64E-01 |
| HFrEF vs Control | male | hsa-miR-151b | 43.5 | -0.078 | 5.24E-01 | 6.71E-01 |
| HFrEF vs Control | male | hsa-miR-30c-1-3p | 43.8 | -0.077 | 5.39E-01 | 6.86E-01 |
| HFrEF vs Control | male | hsa-miR-1229-3p | 13.9 | -0.104 | 5.52E-01 | 6.98E-01 |
| HFrEF vs Control | male | hsa-miR-328-3p | 54.3 | -0.093 | 5.71E-01 | 7.19E-01 |
| HFrEF vs Control | male | hsa-miR-941 | 255.8 | -0.075 | 6.15E-01 | 7.58E-01 |
| HFrEF vs Control | male | hsa-miR-92b-3p | 675.5 | -0.069 | 6.16E-01 | 7.58E-01 |
| HFrEF vs Control | male | hsa-miR-660-5p | 45.4 | 0.067 | 6.14E-01 | 7.58E-01 |
| HFrEF vs Control | male | hsa-miR-501-3p | 32.9 | -0.059 | 6.14E-01 | 7.58E-01 |
| HFrEF vs Control | male | hsa-miR-130b-5p | 14.9 | -0.074 | 6.32E-01 | 7.74E-01 |
| HFrEF vs Control | male | hsa-miR-636 | 84.9 | -0.067 | 6.36E-01 | 7.75E-01 |
| HFrEF vs Control | male | hsa-miR-3200-5p | 25.6 | 0.074 | 6.47E-01 | 7.82E-01 |
| HFrEF vs Control | male | hsa-miR-191-3p | 26.9 | 0.052 | 6.49E-01 | 7.82E-01 |
| HFrEF vs Control | male | hsa-miR-155-5p | 67.8 | -0.056 | 6.63E-01 | 7.95E-01 |
| HFrEF vs Control | male | hsa-miR-145-5p | 17.8 | -0.062 | 6.72E-01 | 8.01E-01 |
| HFrEF vs Control | male | hsa-miR-1304-3p | 82.4 | 0.033 | 6.77E-01 | 8.03E-01 |
| HFrEF vs Control | male | hsa-miR-664a-3p | 9.7 | 0.072 | 6.86E-01 | 8.03E-01 |
| HFrEF vs Control | male | hsa-miR-1270 | 39.3 | -0.064 | 6.82E-01 | 8.03E-01 |
| HFrEF vs Control | male | hsa-miR-24-3p | 345.1 | 0.039 | 6.88E-01 | 8.03E-01 |
| HFrEF vs Control | male | hsa-miR-92b-5p | 19.8 | 0.070 | 6.93E-01 | 8.06E-01 |
| HFrEF vs Control | male | hsa-miR-151a-5p | 12065.9 | 0.040 | 7.14E-01 | 8.26E-01 |
| HFrEF vs Control | male | hsa-miR-30e-3p | 477.3 | 0.055 | 7.18E-01 | 8.26E-01 |
| HFrEF vs Control | male | hsa-miR-628-3p | 17.1 | -0.049 | 7.33E-01 | 8.39E-01 |
| HFrEF vs Control | male | hsa-miR-125b-5p | 243.0 | -0.070 | 7.58E-01 | 8.60E-01 |
| HFrEF vs Control | male | hsa-miR-6511a-3p | 95.9 | -0.046 | 7.59E-01 | 8.60E-01 |
| HFrEF vs Control | male | hsa-miR-3940-3p | 254.9 | -0.056 | 7.78E-01 | 8.74E-01 |
| HFrEF vs Control | male | hsa-miR-26a-5p | 1164.8 | -0.044 | 7.75E-01 | 8.74E-01 |
| HFrEF vs Control | male | hsa-miR-130b-3p | 408.8 | 0.025 | 7.97E-01 | 8.90E-01 |
| HFrEF vs Control | male | hsa-miR-126-5p | 32.2 | -0.081 | 8.05E-01 | 8.95E-01 |
| HFrEF vs Control | male | hsa-miR-3667-5p | 18.2 | 0.042 | 8.34E-01 | 9.18E-01 |
| HFrEF vs Control | male | hsa-miR-501-5p | 14.6 | 0.031 | 8.31E-01 | 9.18E-01 |
| HFrEF vs Control | male | hsa-miR-3605-3p | 1167.7 | -0.025 | 8.46E-01 | 9.27E-01 |
| HFrEF vs Control | male | hsa-miR-6783-3p | 18.0 | -0.031 | 8.60E-01 | 9.38E-01 |
| HFrEF vs Control | male | hsa-miR-183-3p | 132.4 | 0.025 | 8.66E-01 | 9.40E-01 |
| HFrEF vs Control | male | hsa-miR-92a-3p | 19553.8 | -0.017 | 8.83E-01 | 9.53E-01 |
| HFrEF vs Control | male | hsa-miR-130a-3p | 829.3 | 0.020 | 8.89E-01 | 9.56E-01 |
| HFrEF vs Control | male | hsa-miR-1908-5p | 14.7 | 0.025 | 9.16E-01 | 9.59E-01 |
| HFrEF vs Control | male | hsa-miR-5010-5p | 25.3 | 0.023 | 8.97E-01 | 9.59E-01 |
| HFrEF vs Control | male | hsa-miR-21-5p | 16.6 | -0.023 | 9.13E-01 | 9.59E-01 |
| HFrEF vs Control | male | hsa-miR-6877-5p | 12.0 | 0.018 | 9.15E-01 | 9.59E-01 |
| HFrEF vs Control | male | hsa-miR-10399-3p | 13.6 | -0.017 | 9.10E-01 | 9.59E-01 |
| HFrEF vs Control | male | hsa-miR-139-5p | 192.5 | 0.016 | 9.17E-01 | 9.59E-01 |
| HFrEF vs Control | male | hsa-miR-505-5p | 562.2 | -0.010 | 9.35E-01 | 9.73E-01 |
| HFrEF vs Control | male | hsa-miR-197-3p | 203.9 | -0.011 | 9.41E-01 | 9.75E-01 |
| HFrEF vs Control | male | hsa-miR-100-5p | 84.1 | 0.024 | 9.61E-01 | 9.78E-01 |
| HFrEF vs Control | male | hsa-miR-183-5p | 83.4 | 0.010 | 9.57E-01 | 9.78E-01 |
| HFrEF vs Control | male | hsa-miR-589-5p | 175.0 | -0.006 | 9.50E-01 | 9.78E-01 |
| HFrEF vs Control | male | hsa-miR-362-5p | 63.2 | -0.006 | 9.54E-01 | 9.78E-01 |
| HFrEF vs Control | male | hsa-let-7i-3p | 15.6 | -0.006 | 9.68E-01 | 9.81E-01 |
| HFrEF vs Control | male | hsa-miR-326 | 40.2 | 0.004 | 9.79E-01 | 9.88E-01 |
| HFrEF vs Control | male | hsa-let-7f-5p | 3874.2 | 0.002 | 9.90E-01 | 9.93E-01 |
| HFrEF vs Control | male | hsa-miR-10401-3p | 10.0 | 0.002 | 9.93E-01 | 9.93E-01 |

**Supplementary Table S3. DESeq2 results for sex-stratified comparisons of HFpEF or HFrEF vs. Control under the main filter.**

| sex | comparison | term | R^2^ | F | p |
| --- | --- | --- | --- | --- | --- |
| female | HFpEF vs Control | phenotype | 0.020 | 1.547 | 0.0643 |
| female | HFpEF vs Control | age | 0.014 | 1.102 | 0.3169 |
| female | HFpEF vs Control | BMI | 0.009 | 0.701 | 0.8292 |
| male | HFpEF vs Control | phenotype | 0.041 | 3.384 | 0.0004 |
| male | HFpEF vs Control | age | 0.011 | 0.923 | 0.5069 |
| male | HFpEF vs Control | BMI | 0.016 | 1.282 | 0.1874 |
| female | HFrEF vs Control | phenotype | 0.019 | 1.225 | 0.2109 |
| female | HFrEF vs Control | age | 0.010 | 0.646 | 0.8952 |
| female | HFrEF vs Control | BMI | 0.015 | 0.975 | 0.4613 |
| male | HFrEF vs Control | phenotype | 0.043 | 4.141 | 0.0002 |
| male | HFrEF vs Control | age | 0.010 | 0.973 | 0.4504 |
| male | HFrEF vs Control | BMI | 0.013 | 1.247 | 0.2050 |

**Supplementary Table S4. PERMANOVA results for sex-stratified comparisons of HFpEF or HFrEF vs. Control under the main filter.**
